# Structural and photophysical characterization of the small ultra-red fluorescent protein

**DOI:** 10.1038/s41467-023-39776-9

**Published:** 2023-07-12

**Authors:** Atanu Maiti, Cosmo Z. Buffalo, Saumya Saurabh, Felipe Montecinos-Franjola, Justin S. Hachey, William J. Conlon, Geraldine N. Tran, Bakar Hassan, Kylie J. Walters, Mikhail Drobizhev, W. E. Moerner, Partho Ghosh, Hiroshi Matsuo, Roger Y. Tsien, John Y. Lin, Erik A. Rodriguez

**Affiliations:** 1 Cancer Innovation Laboratory, Frederick National Laboratory for Cancer Research, Frederick, MD 21702 USA; 2 Department of Molecular and Cell Biology and California Institute for Quantitative Biosciences, University of California, Berkeley, Berkeley, CA 94720 USA; 3 Department of Chemistry, Stanford University, Stanford, CA 94305 USA; 4 Department of Chemistry, The George Washington University, Washington, DC 20052 USA; 5 Department of Radiology, Boston University, Boston, MA 02118 USA; 6 Protein Processing Section, Center for Structural Biology, Center for Cancer Research, National Cancer Institute, National Institutes of Health, Frederick, MD 21702 USA; 7 Department of Microbiology and Cell Biology, Montana State University, Bozeman, MT 59717 USA; 8 Department of Chemistry and Biochemistry, University of California, San Diego, La Jolla, CA 92093 USA; 9 Howard Hughes Medical Institute, La Jolla, CA 92093 USA; 10 Tasmanian School of Medicine, University of Tasmania, Hobart, Tasmania 7000 Australia; 11 Present Address: Department of Chemistry, New York University, New York, NY 10003 USA

**Keywords:** Single-molecule biophysics, X-ray crystallography, Biological fluorescence

## Abstract

The small Ultra-Red Fluorescent Protein (smURFP) represents a new class of fluorescent protein with exceptional photostability and brightness derived from allophycocyanin in a previous directed evolution. Here, we report the smURFP crystal structure to better understand properties and enable further engineering of improved variants. We compare this structure to the structures of allophycocyanin and smURFP mutants to identify the structural origins of the molecular brightness. We then use a structure-guided approach to develop monomeric smURFP variants that fluoresce with phycocyanobilin but not biliverdin. Furthermore, we measure smURFP photophysical properties necessary for advanced imaging modalities, such as those relevant for two-photon, fluorescence lifetime, and single-molecule imaging. We observe that smURFP has the largest two-photon cross-section measured for a fluorescent protein, and that it produces more photons than organic dyes. Altogether, this study expands our understanding of the smURFP, which will inform future engineering toward optimal FPs compatible with whole organism studies.

## Abstract (web summary)

Researchers determined the three-dimensional structure of the small Ultra-Red Fluorescent Protein (smURFP) to understand its properties and the previous directed evolution process. In addition, they show smURFP fluoresces longer than small molecules.

## Introduction

Fluorescent proteins (FPs) are invaluable biomedical research tools as they enable the visualization of cellular components and monitoring of dynamic processes within living cells and organisms^1,2^. The most widely used FPs, such as the green FP (GFP), form chromophores in a reaction that consumes oxygen and produces a stoichiometric amount of hydrogen peroxide^3^. This limits imaging to aerobic environments tolerant of reactive oxygen species. Furthermore, the chromophores of these FPs have limited spectral properties, such as a fluorescence excitation maximum of ≤610 nm^1,2^. Therefore, they can’t be used for optimal far-red and near-infrared (near-IR) excitation (>610 nm), which are necessary for deep imaging in living animals^2,4,5^.

To overcome these limitations, several far-red and near-IR absorbing FPs have recently been developed from either bacterial phytochromes^6–10^ or cyanobacterial phycobiliproteins^11–13^. These FPs attach biliverdin (BV), a ubiquitous product of heme degradation in vertebrates^14^, as an exogenous chromophore, which avoids the need for oxygen and hydrogen peroxide production while allowing the use of near-IR excitation. However, despite this progress, FPs have lower photostability than spectrally similar small-molecule organic dyes commonly used for super-resolution microscopy^11,15–18^.

Our previous efforts to develop a far-red FP with improved performance relative to other FPs yielded the small Ultra-Red FP (smURFP), which we created from the α-allophycocyanin phycobiliprotein through 12 rounds of directed evolution^11,19^. The resulting smURFP had 20 mutations compared to the parental sequence. Unlike α-allophycocyanin phycobiliprotein which requires a lyase to attach the chromophore, smURFP formed a covalent bond with BV via a cysteine residue without needing a lyase. Furthermore, smURFP is small (16 kD) relative to the GFP (27 kD), and yet its molecular brightness is equivalent to the enhanced GFP (eGFP) with a fluorescence quantum yield (QY) of 18% and a large extinction coefficient (EC) of 180,000 M^−1^ cm^−1^ at 642 nm^1,11,12^. smURFP was successfully incorporated into genetically encoded far-red and near-IR ubiquitination-based cell cycle indicator (FUCCI)^11^ and Förster resonance energy transfer (FRET) kinase sensor^20^. In addition, smURFP is exceptionally stable at room temperature, pH insensitive, easily produced in large quantities, and suitable for ultrasound delivery into corneas^11,12,19,21–23^. These properties make purified smURFP useful for immediate fluorescence imaging without requiring time-consuming protein translation, folding, and chromophore incorporation. Thus far, purified smURFP sensors and probes have been used in numerous applications, for example, to sense BV (detection limit of 0.4 nanomolar)^24^, detect thrombin protease activity (detection limit 0.2 attomolar)^25^, label membrane proteins using sortase-mediated conjugation^26^, label virus-like nanoparticles for imaging animal biodistribution^27^, and for noninvasive in vivo imaging of cancer when incorporated into protein nanoparticles^21^. In addition to these applications, smURFP should be compatible with advanced imaging modalities, such as two-photon, fluorescence lifetime, and single-molecule imaging. However, the characterization of smURFP under these imaging modalities has not been described, thus impeding optimal use and further development.

Additional obstacles to the rational engineering of novel smURFP variants are the lack of structural information and an incomplete understanding of factors that influence the exceptional molecular brightness of smURFP. Although structures of two smURFP mutants (Y56R and Y56F)^28^ have been described, these mutants have diminished QYs and can’t be used to rationalize the optical properties of smURFP.

In this work, we present the crystal structure of smURFP, which enables us to rationalize the outcomes of our previous directed evolution studies and the role of the 20 mutations that this process introduced. The structure reveals that smURFP is a homodimer, and polar, π-π, and hydrophobic interactions mediate dimer formation. Our studies also show that BV incorporation requires dimer formation, as our attempts to design a monomeric smURFP result in a monomeric variant that covalently attaches phycocyanobilin (PCB), a BV-related chromophore found in cyanobacteria and algae, but not BV. Additionally, we conduct in-depth characterizations of smURFP photophysical properties, thus providing a structural and photophysical framework for engineering further smURFP variants suitable for advanced imaging applications.

## Results

### Crystal structure of the apo-smURFP

In our previous studies, we used 12 rounds of directed evolution to obtain smURFP from α-allophycocyanin phycobiliprotein^11^. The resulting smURFP differs from the parental protein by 20 amino acids. Importantly, smURFP exhibited a functional difference as it underwent lyase-independent chromophorylation, i.e., chromophore (BV) attachment. In order to better understand BV attachment and photophysical properties of smURFP, we attempted to determine a crystal structure of the fully chromophorylated smURFP with two BV per homodimer. We previously noticed that the yield of chromophorylation was variable. To understand this further, we quantitatively determined the amount of BV attached to smURFP in high and low protein expression conditions (Supplementary Fig. 1). With high protein expression, smURFP covalently attached 20% BV. In contrast, decreased protein expression improved the yield of BV attachment to 87% (Supplementary Fig. 1b, c). Adding 10-fold excess BV in vitro increased BV attachment further to ≥97% (Supplementary Fig. 1d). We attempted to determine the structure of two BV attached to smURFP, but the dark blue crystals did not diffract well (Supplementary Fig. 1g). Therefore, although we tried to alleviate the structural heterogeneity by saturating the BV sites, other factors, such as chromophore induced changes in crystal packing, may complicate crystallization and further work will be needed to determine the structure of smURFP with two BV.

**Fig. 1:**
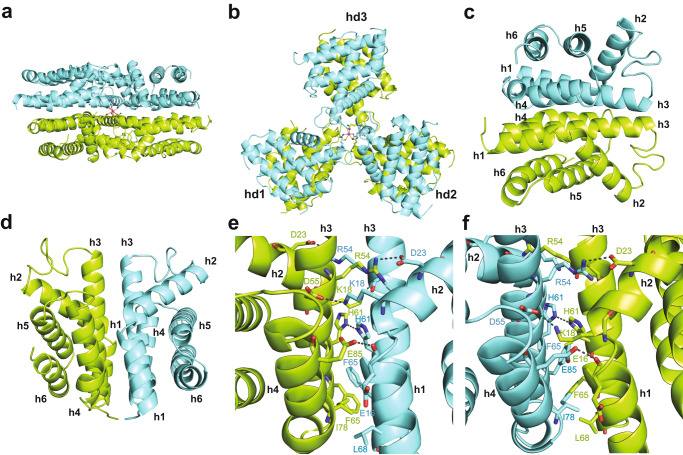
The smURFP crystal structure. The asymmetric unit contains three smURFP homodimers in the *C*222_1_ space group. The smURFP homodimer is cyan and green. At the center is a sodium ion (purple sphere) coordinated by Q46 from each smURFP monomer. **a** A view through the dimeric interface of the three smURFP homodimers. **b** A perpendicular representation of three smURFP homodimers showing a pinwheel-like structure. **c, d** The smURFP monomers each have six α-helices, numbered h1-h6, connected by loops. **c** The two monomers dimerize through helical interactions. **d** Two monomers are shown side-by-side by a 90° rotation on the z-axis followed by a 180° rotation along the x-axis. **e, f** The smURFP dimeric interface is formed by salt bridges and polar, π-π, and hydrophobic interactions. Selected residues are shown as a stick model. **e** Shows residues forming the dimeric interface of smURFP. **f** 180˚ rotation of structure in **e**.

On the other hand, apo-smURFP crystallized from a solution of smURFP with 20% BV attached, enabling us to determine the structure at 2.8 Å resolution (Fig. 1, Supplementary Fig. 2, Supplementary Movie 1). The final refinement of the structure resulted in R-work/R-free of 0.239/0.263, respectively (Supplementary Table 1). We observed that the asymmetric unit contains a hexamer with three smURFP homodimers (Fig. 1a, b). Each smURFP monomer comprises six alpha helixes (h1-h6) connected by loops (Fig. 1c, d). Two monomers form a dimer through hydrogen bonds and polar, π-π, and hydrophobic interactions at the dimeric interface. In total, the smURFP structure contains 252 amino acid hydrogen bonds to stabilize the homodimer (Supplementary Movie 2). The interface is also stabilized by two salt bridges between h2 and h3 (K18, K19 to D55 and D23 to R54) and a π-π interaction between F65 of h3 from each monomer. Hydrophobic interactions also contribute to the interface, most notably L68 of h3 and I78 of h4 of the counterpart (Fig. 1e, f).

**Fig. 2:**
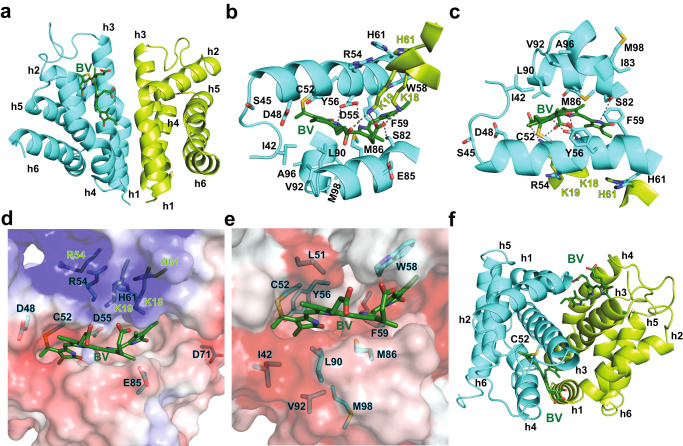
The smURFP interactions with BV. We attached BV to C52 in our smURFP structure using the smURFP Y56R structure^28^ as a template and described in the Methods. **a-e** smURFP with one BV (smURFP+BV). **a** The smURFP protomers are cyan and light green with helices labeled h1-h6 from the N- to C-terminus. **b** An enlarged view of BV in the chromophore pocket. C52 covalently attaches BV to produce fluorescence. All residues ≤5 Å from BV are shown, including amino acids from the adjacent protomer in light green. BV incorporation required dimerization from the structure. **c** 180˚ rotation of structure in **b**. **d** Electrostatic potential surface of charged residues near BV. The charge scale is positive, +3 max in blue, and negative, -3 max in red. **e** Hydrophobicity surface of amino acids near BV. The Eisenberg normalized consensus hydrophobicity scale categorized amino acids^70^; the hydrophobic surface, +1.38 max, is red, and the hydrophilic surface, −2.53 max, is white. **f** smURFP with two BV (smURFP+BV_2_). The smURFP protomers are cyan and light green with helices labeled h1-h6 from the N- to C-terminus. BV is dark green, while nitrogen, oxygen, and sulfur are colored blue, red, and yellow, respectively. Atomic coordinates are available in Supplementary Structures 1,2.

We previously showed C52 covalently attached BV^11^. In our structure, we observed that C52 is located at the edge of h3 within a pocket that binds and subsequently covalently attaches BV. Therefore, we explored BV binding and attachment poses using docking and MD simulations. smURFP covalently attached BV by a thioether bond from C52 to the BV C3_1_ atom (Fig. 2a–c, Supplementary Structure 1). The BV conformation was verified by calculating the free energy of binding in the MD simulation for 20 ns and denaturation of smURFP+BV under acidic conditions (Supplementary Fig. 3). Importantly, the smURFP chromophore pocket is formed by amino acids from both protomers, which agreed with our previous directed evolution studies that indicated that the dimer was required for BV incorporation^11^. D55 plays a central role in holding the BV B and C rings through hydrogen bonding. At the same time, S82 and W58 positioned the D ring through hydrogen bonding and π-π stacking interactions, respectively. The K18 and K19 from the adjacent protomer stabilized the BV by forming salt bridges with the BV carboxylates (Fig. 2b, c). The electrostatic potential surface shows a positively charged pocket to attract the negatively charged BV (Fig. 2d). The pocket is also hydrophobic to stabilize the BV tetrapyrroles (Fig. 2e). From our efforts, we noticed that smURFP accommodated one or two BV chromophores without significant rearrangement of the homodimeric structure (Fig. 2f, Supplementary Movies 3, 4, Supplementary Structure 2). Taken together, the structure of smURFP and the BV docking studies highlight the requirement for dimer formation and identifies a subset of the residues as critical for dimerization and another set as key for chromophorylation.

### Structure-based rationalization of the directed evolution process

To better understand the results of different rounds of the previous directed evolution, as well as rationalize determinants of molecular brightness of the smURFP+BV, we mapped step-wise changes observed during the directed evolution onto the structure of smURFP. As mentioned, 12 rounds of directed evolution starting from α-allophycocyanin accumulated 20 amino acid mutations to create smURFP in our previous study^11^ (Supplementary Figs. 4-6). We examined the smURFP structure determined here to locate these mutations and correlate them with the photophysical properties of the resulting smURFP (Supplementary Table 2). The first mutation that emerged during the directed evolution was the N42I mutation. Based on our structure of smURFP, this mutation is located on top of the chromophore pocket, and it allowed for covalent attachment of PCB but not BV (Supplementary Fig. 4a, Supplementary Table 2). The next set of mutations that emerged during the directed evolution (Y65F, G66C, and V83I) are located at the dimeric interface and are important for stabilizing the dimer. We previously observed that the emergence of these dimer stabilizing mutations coincided with enhanced PCB fluorescence^11^ (Supplementary Fig. 4b, c, Supplementary Table 2).

BV attachment by smURFP required the N42I, G45S, G57R, R61H, Y65F, G66C, V83I, V98M, and Q129K mutations. The N42I, G45S, G57R, R61H, and V83I mutations map onto the chromophore pocket, and suggest that the key change that resulted in BV attachment was a subtle redistribution of hydrophobicity and sterics in the pocket. The V98M and Q129K mutations change the surface charge potential (Supplementary Fig. 4). Furthermore, R61H, Y65F, and G66C are at the dimer interface, in agreement with the observation that BV incorporation requires dimerization (Fig. 2). The Round 5-Clone 2 (R5-2) mutations Y56H and G57R redshifted the fluorescence, which we can now explain based on proximity to BV where the increased steric bulk changes C52 attachment and extends the chromophore conjugation (Supplementary Table 2, Supplementary Figs. 4d, 5a). In this work, we mutated smURFP to create smURFP Y56H and smURFP G57R. These mutations did not redshift the fluorescence, and only smURFP Y56H significantly diminished the QY (Table 1). In our previous directed evolution work, we reverted these two mutations to the original Y56 and G57 on R5-2 by reducing chromophore conjugation and blueshifting the fluorescence using 650 nm excitation light. Rounds 6 and 7 selected R33H and F36L mutations that enhanced the EC and QY. Based on the structure we described here and our MD simulations, these two residues are located within the chromophore pocket loop and stabilize the loop structure (Supplementary Fig. 5b, c, Supplementary Table 2). Therefore, the mutations likely enhanced the QY by chromophore rigidification and improved the EC by extension of the chromophore for increased light absorption.

**Table 1:** Photophysical properties of fluorescent proteins

Fluorescent	Excitation	Emission	Extinction	Quantum	Brightness	Protein
Protein	Maximum	Maximum	Coefficient	Yield	Relative eGFP	Stoichiometry
	(nm)	(nm)	(M^-1^ cm^-1^)	(%)	(%)	
smURFP+BV ^*^	642	672	180,000	18	100	Dimer
R4-1 G57R + BV ^*^	647	674	93,000	9.0	26	>Dimer
R5-2 Y56H G57R + BV ^*^	671	696	71,000	5.3	12	n.d.
smURFP Y56H + BV ^†^	642	672	170,000	8.1	43	n.d.
smURFP G57R + BV ^†^	642	672	170,000	18	94	n.d.
smURFP+*PCB* ^*^	642	666	65,000	7.0	14	Dimer
smURFP H61R ^†^	n.d.	n.d.	n.d.	n.d.	n.d.	Dimer & >Dimer
smURFP F65K + *PCB* ^﻿†^	642	666	23,000	1.5	1.1	Monomer
﻿MsmURFP (smURFP H61R F65K I78K) + *PCB* ^﻿†^	﻿642	666	34,000	2.9	3.0	Monomer
smURFP Y56R + BV ^‡^	672	696	110,000	1.2	4.1	Dimer
smURFP Y56F + BV ^‡^	677	700	73,000	2.7	6.1	Dimer

Values from ^*^ Ref. ^11^, ^†^ this work, and ^‡^ Ref. ^28^. The Methods section describes property determination. Rows 2 & 3 are mutant proteins named Round Number-Clone Number with mutations. n.d. is not determined.

The last rounds in the previously published directed evolution process were aimed at improving expression in *E. coli* to enhance FP stability and chromophore attachment^11^. The structure of smURFP shows the G96A mutation is likely stabilizing the N-terminus of helix 5 (Supplementary Fig. 5d). Furthermore, we observed that several mutations (K9N, E48D, and K118N) increased the stability of the protein by subtly changing the electrostatic potential of the smURFP surface (Supplementary Figs. 5d, 6). Finally, the Y59F mutation rigidized BV in the chromophore pocket for the largest QY and created smURFP (Supplementary Fig. 6d, Supplementary Table 2). Taken together, the smURFP structure allowed us to visualize and rationalize gradual changes that emerged during the 12 rounds of directed evolution to shift the protein from one that required the use of a lyase to attach the chromophore, to one that does so in an enzyme-independent manner. Moreover, the mutations also resulted in expanding the chromophore preference to include PCB and BV, as well as improved photophysical properties (i.e., increased EC and QY) and the stabilization of the dimer required for BV attachment.

### Oligomerization of the smURFP in living cells

Although our structural analysis produced evidence for dimer formation and the previous directed evolution studies indicated that dimerization was required for BV attachment, we wanted to confirm that smURFP dimerizes in living cells. To investigate smURFP oligomerization, we employed the Organized Smooth Endoplasmic Reticulum (OSER) assay, an established method for detecting oligomerization of FP fusions in living cells^29^. For the purpose of the assay, we fused smURFP to the first 29 amino acids of the N-terminus transmembrane domain of cytochrome p450 (CytERM), which ensures expression on the cytoplasmic face of the endoplasmic reticulum (ER). The OSER assay then detects whether the FPs on opposing ER membranes associate with each other by quantifying the number of OSER whorl structures and determining the ratio of the mean fluorescence intensity (MFI) of the OSER structure divided by the MFI of the nuclear envelope^29^. The OSER assay of smURFP was compared to monomeric GFP (mGFP) as a control after 24 and 48 h (Supplementary Fig. 7). We observed 17% and 21% of cells expressing CytERM-mGFP showed abnormal OSER whorl structures after 24 and 48 h, respectively. In the case of CytERM-smURFP, we observed abnormal OSER whorl structures in 68 and 75% of cells after 24 and 48 h, respectively (Supplementary Fig. 7a-d). CytERM-mGFP showed an average ratio of <2.3 at both time points, indicative of no oligomerization (Supplementary Fig. 7e). In contrast, CytERM-smURFP average ratio was 4.8 ± 0.2 after 24 h and decreased to 3.7 ± 0.3 after 48 h due to the increased MFI of the nuclear envelope. These values indicate that smURFP fusions expressed in cells form oligomers under the conditions of the OSER assay, suggesting that our structural analysis likely captured the functionally relevant form of smURFP.

### Initial design and characterization of monomeric smURFP

**Fig. 3:**
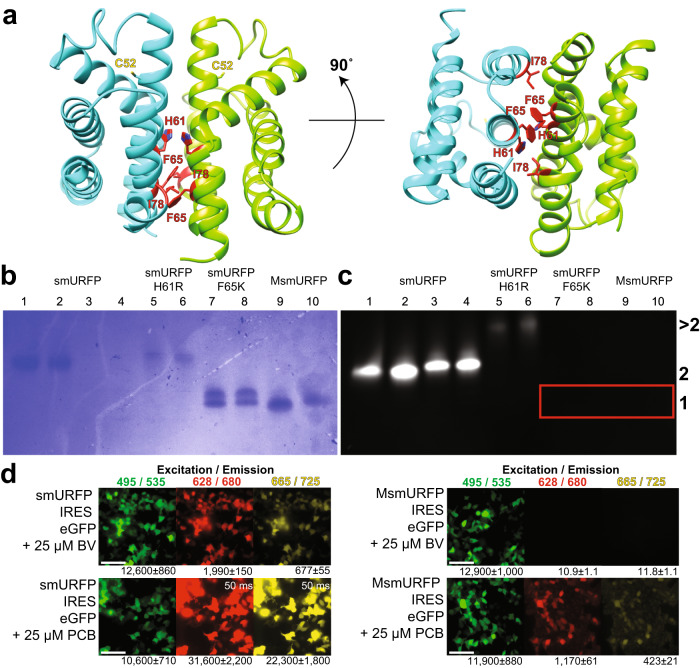
Initial design and characterization of monomeric smURFP. **a** The dimeric interface of the smURFP with H61, F65, and I78 in red. The side view (left) and 90˚ rotation for the bottom view (right). smURFP was mutated to create three variants: smURFP H61R, smURFP F65K, and smURFP H61R, F65K, and I78K (named MsmURFP). A native gel determined the oligomerization (*n* = 1 gel of two independent protein purifications). **b** Coomassie stain of total protein. **c** Far-red fluorescence verified chromophore attachment. smURFP is a fluorescent homodimer and is used as a size reference. smURFP H61R is a dimer and a larger oligomer. smURFP F65K and MsmURFP ran as lower MW bands and are non-fluorescent without BV (red box). Odd and even lanes are without and with 100 mM DTT, respectively. The numbers on the right are oligomerization, where >2 is larger than a dimer, 2 is a dimer, and 1 is a monomer. **d** HEK293A cells transiently expressed smURFP or MsmURFP IRES eGFP for 45 h following chromophore incubation for 3 h. eGFP verifies the transfection of DNA. smURFP shows far-red fluorescence with the addition of BV and PCB. MsmURFP fluoresces only with the addition of PCB, which is not present in human cells. The numbers below images are the MFI (*n* = 40 cells) ± standard error of the mean (SEM). The scale bars are 100 μm.

One possible obstacle to using smURFP as a fluorescent imaging probe is the obligatory dimer form. To examine whether the system could be further simplified into a monomeric smURFP, we used our smURFP homodimeric structure to engineer monomeric variants of smURFP. We focused on mutations at H61, F65, and I78, as our structure points to their key role in mediating dimer formation (Figs. 1, 3a, Supplementary Movie 5). We mutated these residues into large, positively charged amino acids to break the homodimer through steric and charge repulsion, thus generating smURFP H61R, smURFP F65K, and smURFP H61R, F65K, and I78K (named MsmURFP). We purified the mutant proteins from *E. coli* with BV production and analyzed them with a native gel to determine protein stoichiometry and chromophore attachment (Fig. 3b, c). We observed that smURFP+BV migrated as a highly fluorescent homodimer, which we used as a size reference. In contrast, smURFP H61R formed a non-fluorescent homodimer and dimly fluorescent oligomer larger than a dimer. smURFP F65K ran as two non-fluorescent protein bands smaller than a dimer. We used a denaturing SDS-PAGE gel to confirm that these doublet bands were not caused by proteins of different lengths (Supplementary Fig. 8). The MsmURFP ran as a single, monomeric protein band and was non-fluorescent.

Given that BV attachment requires dimerization (Fig. 2), and the PCB incorporation of our initial FP variant does not (Supplementary Fig. 4a), we hypothesized that monomeric variants could covalently attach PCB. To verify the monomeric variants were still functional, we added PCB in vitro. We observed that MsmURFP+PCB was brighter than smURFP F65K + PCB (Table 1). MsmURFP was further tested for incorporation of PCB in human embryonic kidney (HEK293A) cells. smURFP and MsmURFP were placed before an Internal Ribosomal Entry Site (IRES) with eGFP to create a single mRNA that produces both FPs (Fig. 3d). The smURFP+PCB MFI (31,600) was greater than smURFP+BV MFI (1990) due to methylation of PCB carboxylates that improves membrane permeability^11^. MsmURFP showed fluorescence only with the addition of PCB (MFI = 1170). No fluorescence was seen above the background for MsmURFP with 25 μM BV or 5 μM BVMe_2_ (MFI = 10.9 and 25.3, respectively). Attempts to perform the OSER assay or fusion to α-tubulin with the MsmURFP+PCB did not produce adequate fluorescence. Taken together, these studies illustrate that the monomeric smURFP variant MsmURFP lost the ability to incorporate BV, further confirming that dimer formation was a critical requirement. However, MsmURFP is functional and covalently attaches PCB to generate fluorescence, suggesting that further directed evolution of MsmURFP may represent a viable strategy for developing monomeric smURFPs that incorporate BV without dimerization.

### Characterization of the smURFP in advanced microscopy techniques

Historically, small-molecule organic dyes have been the probes of choice for super-resolution imaging and advanced optical microscopy studies due to their high photostability and brightness. We previously developed smURFP as a photostable and bright FP and proposed that it can be an alternative to organic dyes. However, the performance of smURFP under advanced microscopy techniques has not been previously characterized. Here, we examined smURFP as a probe for two-photon, fluorescence lifetime, and single-molecule imaging.

**Fig. 4:**
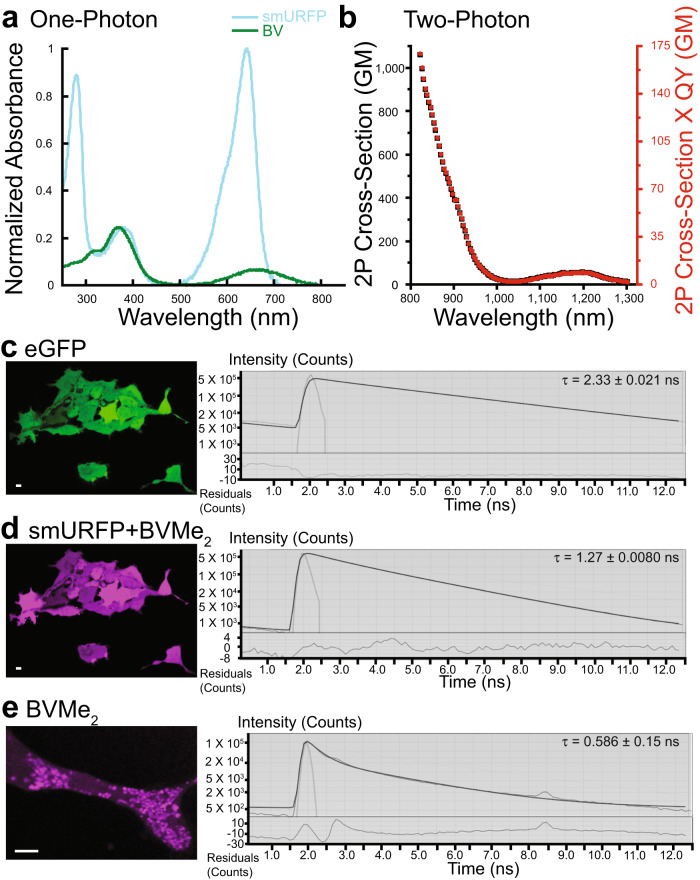
Photophysical characterization of the smURFP two-photon cross-section and FLIM. **a** One-photon absorption spectra of free BV (green) and smURFP+BV (blue). BV shows characteristic Soret and broad Q bands at 380 and 670 nm, respectively. smURFP+BV Soret and narrow Q bands are at 383 and 642 nm, respectively. Protein absorbance is at 280 nm. Covalent attachment of BV to smURFP enhances the EC to 180,000 M^-1^ cm^-1^ and causes increased absorbance at 642 nm. **b** The smURFP maximum two-photon cross-section (black) is seen at 820 nm with 1,060 GM. A second maximum is 1,196 nm with 60 GM. The two-photon brightness is in red. **c–e** FLIM of eGFP, smURFP+BVMe_2_, and free BVMe_2_ in HEK293A cells. **c, d** HEK293A cells stably expressing eGFP and smURFP were incubated with 1 μM BVMe_2_ for 24 h. eGFP and smURFP+BVMe_2_ have fluorescence lifetimes of 2.33 and 1.27 ns, respectively. **e** Wild-type HEK293A cells were incubated with 10 μM BVMe_2_ for 24 h. BVMe_2_ fluorescence appeared in organelles or vesicles with a fluorescence lifetime of 0.586 ns. The right panel shows the exponential fluorescence decay and fits, with the mean fluorescence lifetime ± standard deviation (SD) in the upper right corner for *n* = 10 cells. The scale bars are 10 μm.

The one-photon absorption spectra of smURFP+BV and BV are shown in Fig. 4a. The smURFP scaffold creates a narrow Q-band absorbance with BV. The large smURFP+BV EC (180,000 M^−1^ cm^−1^) should translate to a large two-photon cross-section for deep, multi-photon imaging. We measured the two-photon cross-sections of smURFP as 60 GM at 1196 nm and 1060 GM at 820 nm (Fig. 4b). The two-photon absorption at 1196 nm is blueshifted relative to the doubled one-photon peak wavelength of 1284 nm due to the vibronic enhancement effect^30^. smURFP has a narrower Q band compared to near-IR FPs (iRFPs)^8^, allowing excitation at shorter wavelengths to achieve double-resonance conditions^31–33^. Thus, the smURFP two-photon cross-section of 1060 GM at 820 nm is the largest recorded for an FP (Fig. 4b), suggesting that its performance in two-photon imaging experiments would be comparable to organic dyes.

With respect to fluorescence lifetime imaging (FLIM), fluorescence lifetime is sensitive to the environment and is proportional to the QY^34,35^. The BV QY is 0.013%, and the smURFP covalent attachment of BV enhances the QY 1400-fold to 18%^11^. As a part of the initial characterization, we measured the fluorescence lifetime of purified smURFP+BV as 1.40 ± 0.0040 ns. Given that BV is weakly membrane-permeant and degraded into bilirubin (BR) inside cells, we previously overcame these issues by using a more hydrophobic biliverdin dimethyl ester, BVMe_2_^2,11^. Thus, we performed FLIM of BVMe_2_ free and covalently attached to smURFP inside living cells. We measured the fluorescence lifetime of eGFP and smURFP expressed from the same mRNA in HEK293A cells (Fig. 4c, d). eGFP showed a fluorescence lifetime of 2.33 ± 0.021 ns, which was similar to the reported lifetime of 2.4 ns^36^. smURFP+BVMe_2_ has a fluorescence lifetime of 1.27 ± 0.0080 ns and is comparable to our in vitro measurement of 1.40 ns. Wild-type HEK293A cells were incubated with 10 μM BVMe_2_, and free BVMe_2_ has a fluorescence lifetime of 0.586 ± 0.15 ns. The eGFP and smURFP fluoresce in the entire cell, while the free BVMe_2_ is in subcellular organelles or vesicles (Fig. 4e). Despite the smURFP+BVMe_2_ and free BVMe_2_ having similar fluorescence excitation and emission, we separated the two chromophores using FLIM to identify the cellular localization.

**Fig. 5:**
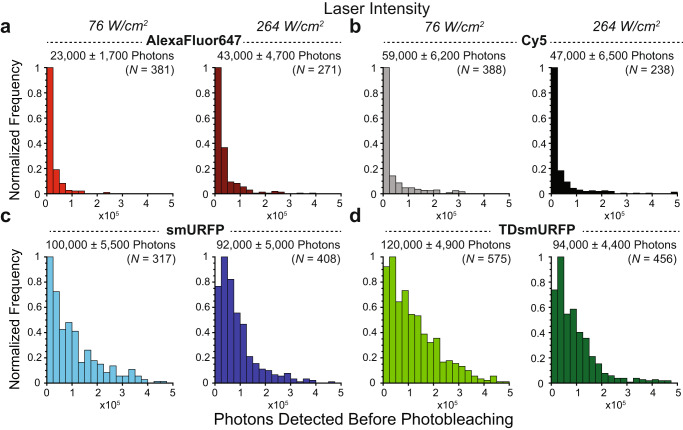
Single-molecule photons detected before photobleaching. Small-molecule organic dyes, AlexaFluor647 and Cy5, were compared to smURFP+BV and TDsmURFP+BV. Single molecules doped in PVA were excited with a 638 nm laser at 76 or 264 W/cm^2^. **a** An average of 23,000 or 43,000 photons were detected before photobleaching from AlexaFluor647 when excited by 76 or 264 W/cm^2^ intensity, respectively. **b** An average of 59,000 or 47,000 photons were detected from Cy5. **c-d** An average of 100,000 and 120,000 or 92,000 and 94,000 photons were detected before photobleaching from (**c)** smURFP+BV or (**d**) TDsmURFP+BV, respectively. The average number of photons detected before photobleaching ± standard error of the mean (SEM) and the number of single molecules measured (*n*) is above each graph.

Lastly, given the high molecular brightness of smURFP under one- and two-photon excitation, we wanted to test single-molecule photostability for long-term imaging. Single-molecule fluorescence was imaged until photobleaching to count the average number of photons detected when excited by a 638 nm laser at 76 and 264 W/cm^2^ intensities (Fig. 5). We compared smURFP and Tandem Dimer smURFP (TDsmURFP), where the homodimer was linked with 23 amino acids^11^, to spectrally similar organic dyes AlexaFluor647 and Cy5. 23,000 and 59,000 or 43,000 and 47,000 photons were detected before photobleaching for AlexaFluor647 or Cy5 when excited by 76 or 264 W/cm^2^ intensity, respectively. For smURFP+BV, we detected 100,000 or 92,000 photons before photobleaching when excited by 76 or 264 W/cm^2^ intensity, respectively. Similarly, 120,000 or 94,000 photons were detected before TDsmURFP+BV photobleaching when excited by 76 or 264 W/cm^2^ intensity, respectively. Therefore, our results indicate that smURFP+BV is twice as photostable as AlexaFluor647 and Cy5. Collectively, these studies highlight that due to its remarkable photophysical properties and photostability, smURFP represents a protein-based probe that could substitute for organic dyes in advanced microscopy applications.

## Discussion

Here, we describe the structure of apo-smURFP, and use this novel structural information as a molecular framework to rationalize the results of our previous directed evolution^11^ and explain the origins of the molecular brightness. The crystal structure also allowed us to design monomeric smURFP variants by helping us identify key residues that mediate smURFP dimer formation and suggesting ways to mutate them to disrupt the dimer yet preserve the protomer structure. Although the resulting monomeric variant MsmURFP (smURFP H61R F65K I78K) incorporated PCB, it lost BV attachment that requires a homodimer. MsmURFP may find future use as a fluorescent PCB sensor to quantify PCB availability for optogenetic tools^37^. Therefore, MsmURFP represents a starting point for further protein engineering to create monomeric FPs with PCB covalently or non-covalently attached for far-red and near-IR fluorescence, as shown by the FP ApcE2^38^. Additionally, MsmURFP can be used as a starting template to create FPs that incorporate BV without dimerization. Finally, we validated that smURFP is a useful molecular probe for advanced microscopic applications, opening opportunities for broader use of this FP due to the large two-photon cross-section and high photostability comparable to organic dyes.

To place the structure of smURFP in context, we compared it to the structure of allophycocyanin from the marine cyanobacterium *Phormidium* sp. A09DM allophycocyanin (PDB ID: 4RMP)^39^, a protein closely related to the *Trichodesmium erythraeum* α-allophycocyanin that served as a parental sequence for our previously described directed evolution work that yielded smURFP^11^ (Supplementary Fig. 9). Overall, we noticed a high degree of similarity between protomer structures that displayed secondary structure conservation (Supplementary Fig. 10), showing that directed evolution, for the most part, preserved the secondary structure. However, we also observed numerous differences, especially in the hexamer observed in the crystal and the dimeric interface. Whereas allophycocyanin hexamer is composed of three heterodimers of αβ subunits that partially overlap side-by-side to form a hollow disc, the three homodimers of smURFP form a smaller pinwheel-like structure (Supplementary Fig. 11). Additionally, the αβ-heterodimeric interface is smaller compared to the smURFP dimeric interface and mediated by a smaller number of interactions (Supplementary Fig. 12). Major differences are also observed between the two chromophore pockets. The α-allophycocyanin PCB pocket is open to allow lyase binding for covalent attachment of PCB (Supplementary Fig. 13). On the other hand, smURFP attaches BV without a lyase by using amino acids from both protomers to form the chromophore pocket (Fig. 2). Taken together, the structure of smURFP displayed important, functionally relevant differences when compared to the structure of the parental allophycocyanin.

Our analysis also included mapping mutations that emerged during the previously published directed evolution onto the now available structure of smURFP (Supplementary Figs. 4-6). This process enabled us to visualize gradual changes that led from the parental α-allophycocyanin, a protein that forms a covalent bond with PCB in a reaction catalyzed by a lyase, to smURFP that attaches BV in a dimer-dependent and lyase-independent manner. The amino acid changes favored by the directed evolution included the accumulation of mutations that stabilized the smURFP dimer, as well as BV rigidification to enhance the QY.

To better understand determinants of the molecular brightness, we also compared our smURFP structure (PDB ID: 7UQA) with the previously reported crystal structures of smURFP Y56R (PDB ID: 6FZN)^28^ and smURFP Y56F (PDB ID: 6FZO)^28^, two smURFP mutants with diminished QYs (1.2% and 2.7%, respectively^28^, compared to 18% for smURFP^11^, Supplementary Figs. 14, 15 and Table 1). We observed that both protomers of smURFP and the smURFP Y56F mutant align well (Supplementary Fig. 15b). However when smURFP Y56R is aligned to smURFP, a protomer is rotated 180° and breaks symmetry (Supplementary Fig. 15c). The comparison of the chromophore pockets revealed that the pocket in the smURFP Y56F mutant was larger at 663 Å^3^ with disordered loops compared to smURFP, which forms a smaller pocket (576 Å^3^) with a compact and rigid secondary structure (Supplementary Fig. 15b, Supplementary Table 3). This suggests that the superior photophysical properties of smURFP over the smURFP Y56F mutant may, in part, be due to the presence of the rigid and preformed BV pocket. To compare smURFP Y56R and smURFP, we rotated the smURFP Y56R protomer 180° and observed that the loops surrounding the chromophore are closely aligned (Supplementary Fig. 15d, e). Alignment of all the three structures by the α-helix containing residues 52 and 56 shows the 56 amino acid side chain is <4 Å from the BV attachment by C52 (Supplementary Fig. 15f-h, Supplementary Movie 6). This suggests that residue 56 influences chromophore photophysical properties by electron donating/withdrawal and rigidification. Moreover, the mutations at position 56 result in drastic changes in the total protein volumes (Supplementary Table 3), with smURFP having the smallest overall protein volume (31.4 nm^3^), which should rigidize BV, thus giving the highest QY. Altogether, those striking structural deviations between smURFP and the mutants explain the differences in photophysical properties. Because we report the crystal structure of the apo-smURFP, future structures including the chromophore will be necessary to validate the hypotheses herein advanced.

In addition to determining the smURFP structure, we measured photophysical properties for advanced imaging modalities. We tested the performance of smURFP in two-photon, fluorescence lifetime, and single-molecule imaging applications. In fluorescence comparisons, smURFP outperformed other FPs, organic dyes, or both. For example, smURFP has the largest two-photon cross-section measured for an FP and superior two-photon brightness. For comparison to other FPs and organic dyes, iRFPs have two-photon brightness of 13−35 and 1.9–8.7 GM from 880 to 900 and 1200 to 1280 nm, respectively^8^, and AlexaFluor647 and Cy5 have two-photon brightness of ~210 and ~45 GM at 800 and 1230 nm, respectively^40,41^. Thus, with the two-photon brightness of 190 and 11 GM at 820 and 1196 nm, respectively, the smURFP 820 nm two-photon brightness is greater or equivalent to fluorophores used for multi-photon imaging. Therefore, smURFP is a valuable FP for incorporation into future probes and sensors to improve in vivo imaging. Furthermore, we differentiated free BVMe_2_ and BVMe_2_ attached to smURFP by FLIM in living cells. Finally, we performed single-molecule imaging to determine the photostability of smURFP relative to organic dyes. In general, small-molecule organic dyes are more photostable than FPs^15–18^. However, our results show that smURFP+BV is twice as photostable as AlexaFluor647 and Cy5, suggesting that smURFP will find use in long-term imaging and super-resolution microscopy.

In conclusion, our study provides a structural basis for rationalizing the directed evolution and photophysical properties of smURFP. We demonstrate this remarkable FP is comparable to or outperforms tested FPs and organic dyes, thus opening the doors to using smURFP for advanced microscopy techniques. Further work is needed to develop optimized monomeric forms of the protein. We expect that dimeric and monomeric smURFPs will significantly impact our ability to image deeply in tissues and organisms.

## Methods

### Chemicals and reagents

Unless noted, chemicals and reagents were from MilliporeSigma, ThermoFisher, or Alfa Aesar. BV hydrochloride (B655-9) and BVMe_2_ (B610-9) were from Frontier Scientific. PCB was purified from *Spirulina*^42^. 5 mM chromophore stocks were dissolved in DMSO, aliquoted, and stored at −20 °C.

### FP purification

smURFP or variants in a modified pBAD vector with a C-terminal hexahistidine-tag, bacterial ribosomal binding site, and *Synechocystis* HO-1^7,43,44^ for BV production (Addgene 80341) transformed fresh TOP10 *E. coli* (ThermoFisher C404006). *E. coli* grew in LB + 0.02% Arabinose (MilliporeSigma A91906-25G-A) + 50 µg/ml ampicillin (MilliporeSigma A1593-25G) at 37 °C for 72 h. To increase BV incorporation in smURFP, the same plasmid was transformed in TOP10 *E. coli* and grown in LB + 0.002% arabinose to reduce smURFP expression + 50 µg/ml ampicillin at 37 °C for 72 h. Cells were pelleted by 5422 × *g* centrifugation at 4 °C and lysed with B-PER (ThermoFisher 90084) + DNAseI (ThermoFisher EN0521) at RT with shaking for 1 h. The soluble fraction was loaded on a Nickel-NTA Resin (G-Biosciences 786940), washed with 50 mM Tris (pH 7.5) + 300 mM NaCl + 10 mM imidazole, eluted with 50 mM Tris (pH 8) + 300 mM NaCl + 200 mM imidazole, and buffered exchanged using PD-10 Desalting Columns (GE Healthcare 17-0851-01) into 10 mM Tris (pH 8). 400 µM BV was added to 40 µM smURFP purified from *E. coli* induced with 0.002% Arabinose in 1X PBS + 10% fetal bovine serum (FBS) with 250 RPM shaking for 24 h at 37 °C. Nickel-NTA purification removed unattached BV. Denaturing PAGE gel and Zinc blot screened BV attachment (Supplementary Fig. 1a). 1.1, 2.2, 4.4, and 8.8 µg of smURFP+BV (0.02% Arabinose in LB) were loaded in lanes 2, 3, 4, and 5, respectively, with Precision Plus Protein Dual Color Standards (Bio-Rad 1610374) in lanes 1 on a NuPAGE Novex 4-12% Bis-Tris gel. NuPAGE MOPS SDS running buffer ran the SDS-denaturing PAGE gel. To image protein with BV covalently attached, 100 ml 1 M Zn(OAc)_2_ was added to the gel and incubated at RT with shaking for 30 min. A UVP ChemStudio PLUS Darkroom with Motorized Lift and OptiChemi 615 3.2 MP Cooled CCD Camera (Analytik Jena) with EX/EM = 685/710 LP nm imaged fluorescence of BV + Zinc, as described^44^. The gel was stained with Coomassie Brilliant Blue to visualize total protein.

### Mass spectrometry quantification of chromophore attachment

The mass of smURFP with and without BV was determined using liquid chromatography-mass spectrometry (LC-MS). The purified protein was injected into the electrospray interface on a ThermoFisher Orbitrap XL (Supplementary Fig. 1b-d). An Agilent Technologies Liquid Chromatography 1100LC used a solvent system of Solvent A: 2% acetonitrile with 0.1% formic acid in water and Solvent B: 90% acetonitrile with 0.1% formic acid in water. The flow rate was 80 µl/min with a gradient from 18% Solvent A to 100% Solvent B in 2.4 min. smURFP eluted by 10 min from a 1 mm inner diameter, 50 mm PLRP-S C_18_ column (Agilent Technologies). The Orbitrap XL electrospray interface had a sheath gas flow rate of 34 at 275 °C. Capillary and test tube lenses were 39 and 140 V, respectively. Mass spectra were collected in the ion trap in Fourier transform profile mode, with a resolution of 60,000 from 500 to 1800 mass-to-charge units. The mass spectra were deconvolved using ProMassCalc or Extract_MSN in Xcalibur V. 2.2 software (ThermoFisher). We obtained each mass spectra from a single injection (*n* = 1 protein purification) of purified FP ( >30 µM), and each sample was repeated with three separate injections.

### Crystallization and X-ray diffraction data collection

smURFP was concentrated using an Amicon 10,000 MWCO filter (MilliporeSigma UFC8010) to ∼20 mg/ml and immediately used to avoid precipitation. The screen used 1,440 conditions with a 1:1, 2:1, and 1:2 reservoir solution:protein ratio in 200 nl drops over 480 reservoir solutions with the Mosquito® Crystal (SPT Labtech). Crystals were formed by the hanging-drop vapor diffusion method. Scaling up, smURFP crystallized at 20 °C by mixing 2 µl 625 µM smURFP+BV with 1 µl of the reservoir solution, 4 M Na nitrate + 0.1 M NaOAc (pH 4.6) (SaltRx™ 1 library (Hampton Research HR2-107)). Crystals (Supplementary Fig. 1e-f) were transferred to the reservoir solution with 20% glycerol, mounted in fiber loops, and flash-cooled in liquid N_2_ for cryopreservation. Diffraction data were collected from smURFP crystals under cryogenic conditions at the Advanced Photon Source (APS at Argonne National Laboratory) beamline 24-ID-C (NE-CAT Center for Advanced Macromolecular Crystallography, Cornell University).

smURFP with ≥97% BV was crystallized under similar conditions. Small, dark blue crystals formed (Supplementary Fig. 1g) but did not provide sufficient diffraction data for structure determination. smURFP with ≥97% BV was screened with the SaltRx™ 1 library (Hampton Research HR2-107). The 1,440 conditions did not yield crystals that diffracted well enough for structure determination.

### Structure determination, refinement, and validation

The 2.80 Å crystal diffraction data were indexed, integrated, and scaled using HKL-2000 V. 715.5^45^. The smURFP crystal belongs to the space group *C*222_1_. Molecular replacement was performed using Phaser V. 2.8.2 in the CCP4 program suite V. 7.0.051^46^. A molecular replacement solution was obtained using Chain E from the crystal structure of allophycocyanin from *Synechocystis* sp. *PCC 6803* (PDB ID: 4PO5) as the search model^47^. The asymmetric unit comprised six smURFP protomers or three homodimers. Model building and refinement of smURFP were manually performed using the programs Coot V. 0.8.9.2^48^ and Phenix V. 1.12_2829^49,50^, respectively. The refined, final model had R_work_ of 23.94% and R_free_ of 26.34%. MolProbity V. 4.02b-467^51^ validated the smURFP structure. Statistics of Ramachandran analysis yielded 95.88% of the residues in the favored regions, 3.86% in the allowed regions, and 0.26% of residues in the disallowed region. Atomic coordinates and structure factors are in the Protein Data Bank under accession code 7UQA.

### Generation of smURFP + BV and smURFP + BV_2_ models

We placed Biliverdin IXα (BV, Ligand ID: BLA [https://www.rcsb.org/ligand/BLA]) into the electron density of the smURFP Y56R (PDB ID: 6FZN) structure^28^, and refined the structure to validate the BV position. Then, we attached BV at the C3_1_ atom to C52 of our smURFP structure using the refined smURFP Y56R + BV structure. We superimposed smURFP chain E or F to smURFP Y56R chain A. The Schrödinger V. 2021.2 and Desmond V. 6.6 software packages performed MD simulations using our smURFP to add one and two BV chromophores^52^. Prime added missing sidechains and loops, and PROPKA3 determined the protonation states of ionizable sidechains^53–55^. Both termini were capped, and the OPLS4 forcefield minimized hydrogens^56^. The resulting system was placed in a rhombic dodecahedron periodic boundary condition and solvated by the TIP3P water model^57^. Counterions neutralized the system by adding counterions to a total ionic strength of 150 mM to mimic physiological conditions. The Desmond NVT and NPT ensemble equilibrated the system with 50 kcal/mol restrains on heavy atoms for 12 ps. NPT ensemble performed a final unrestrained equilibration for 24 ps. During the MD simulations, we placed 50 kcal/mol position restraints on the ligand. Simulations were performed with 2 fs timesteps for 20 ns. The RESPA integrator in the NPT ensemble was 300 K^58^. The Nosé–Hoover thermostat and Martyna-Tobias-Klein (MTK) barostat maintained temperature and pressure^59,60^. We calculated short-range non-bonded interactions with a 9 Å cutoff, and particle mesh Ewald (PME) calculated long-range electrostatic interactions^61^. Atomic coordinates are available as Supplementary Structures 1, 2 in the Source Data file.

### Crystal structure representations and protein sequence alignments

BV was removed from the structure of smURFP Y56R (PDB ID: 6FZN), and ^3^V: Voss Volume Voxelator V. 1.3^62^ created internal cavities for smURFP, smURFP Y56F, and smURFP Y56R. UCSF Chimera Software V. 1.16 or X 1.5^63^ measured chromophore cavities and protein volume, created Figs. 1, 2a–c, f, 3a, and Supplementary Figs. 4-6, 10-13, 15, and made Supplementary Movies 1-6. PyMOL V. 1.8.6.2 calculated the electrostatic potential and hydrophobicity surfaces using the APBS plugin and the Color_h script, respectively (Fig. 2d, e). EMBL-EBI Clustal Omega V. 1.2.4 (https://www.ebi.ac.uk/Tools/msa/clustalo/)^64^ aligned protein sequences, and MView V. 1.63^65^ determined the consensus sequence and percent identity in Supplementary Figs. 9, 14. The FP net charge was calculated with Innovagen Protein Calculator (http://pepcalc.com/protein-calculator.php) in Supplementary Table 2.

### smURFP + BV acid denaturation

150 µl of 27 µM smURFP+BV was denatured with 900 µl of 8 M guanidinium chloride (ThermoFisher, 24115) and 10 µl of 37% hydrochloric acid (ThermoFisher, 258148). Denatured samples were incubated for 5 min at room temperature with and without illumination at 600 or 650 nm (150 W halogen Type EKE, UVP eLITE MultiSpectral Light Source, Analytik Jena). Absorbance spectroscopy on the Jasco V-770 UV/Visible Spectrophotometer characterized samples. Samples spent <10 min in acid to avoid side reactions.

### smURFP Y56H and smURFP G57R bacterial expression plasmids

QuikChange Lightning Site-Directed Mutagenesis Kit (Agilent 210518) mutated pBAD smURFP RBS HO-1 (Addgene 80341) to create smURFP Y56H and smURFP G57R for protein purification from *E. coli*. Sanger sequencing (Genewiz) verified the mutations.

pBAD smURFP Y56H RBS HO-1 used a QuikChange with the 5’-3’ primers (Integrated DNA Technologies) GCACTGTGCCTGCGTGACCACGGCTGGTTTCTGCACCTGATCACG and CGTGATCAGGTGCAGAAACCAGCCGTGGTCACGCAGGCACAGTGC.

pBAD smURFP G57R RBS HO-1 used a QuikChange with the 5’-3’ primers

GCACTGTGCCTGCGTGACTACCGCTGGTTTCTGCACCTGATCACG and

CGTGATCAGGTGCAGAAACCAGCGGTAGTCACGCAGGCACAGTGC.

### smURFP H61R, smURFP F65K, and MsmURFP expression plasmids

QuikChange mutated pBAD smURFP RBS HO-1 (Addgene 80341) to create smURFP H61R, smURFP F65K, and smURFP H61R, F65K, and I78K (MsmURFP) for protein purification from *E. coli*. Sanger sequencing verified the mutations.

pBAD smURFP H61R RBS HO-1 used a QuikChange with the 5’-3’ primers CTGCGTGACTACGGCTGGTTTCTGCGCCTGATCACGTTCTGTCTGCTGGCTGGTGATAAAGG and CCTTTATCACCAGCCAGCAGACAGAACGTGATCAGGCGCAGAAACCAGCCGTAGTCACGCAG.

pBAD smURFP F65K RBS HO-1 used a QuikChange with the 5’-3’ primers CTGCGTGACTACGGCTGGTTTCTGCACCTGATCACGAAATGTCTGCTGGCTGGTGATAAAGG and CCTTTATCACCAGCCAGCAGACATTTCGTGATCAGGTGCAGAAACCAGCCGTAGTCACGCAG.

pBAD MsmURFP RBS HO-1 used sequential QuikChange reactions. The first QuikChange used the 5’-3’ primers CTGCGTGACTACGGCTGGTTTCTGCGCCTGATCACGAAATGTCTGCTGGCTGGTGATAAAGG and CCTTTATCACCAGCCAGCAGACATTTCGTGATCAGGCGCAGAAACCAGCCGTAGTCACGCAG to introduce the H61R and F65K mutations. The second QuikChange added I78K mutation with the 5’-3’ primers

GTCCTATTGAATCCAAAGGTCTAATTAGCATACGCG and CGCGTATGCTAATTAGACCTTTGGATTCAATAGGAC.

pcDNA3 smURFP IRES eGFP (Addgene 80343) created the MsmURFP with QuikChange. pcDNA3 MsmURFP IRES eGFP used two sequential QuikChange reactions. The first QuikChange used the 5’-3’ primers CTGCGAGATTACGGCTGGTTCCTGCGGCTGATCACCAAATGTCTGCTGGCCGGAGATAAG and CTTATCTCCGGCCAGCAGACATTTGGTGATCAGCCGCAGGAACCAGCCGTAATCTCGCAG to introduce the H61R and F65K mutations. The second QuikChange added I78K mutation with the 5’-3’ primers

GGGCCCCATCGAGTCTAAAGGGCTGATCAGTATTCGAG and CTCGAATACTGATCAGCCCTTTAGACTCGATGGGGCCC.

### Oligomerization characterization

Top10 *E. coli* with 0.02% Arabinose induction expressed smURFP, smURFP H61R, smURFP F65K, and MsmURFP. We purified protein as described in the **FP purification**. A NativePAGE Novex Bis-Tris Gel System determined oligomerization with homodimeric smURFP used as a size reference. 0.27 μg purified protein with 2.5 μl 4X NativePAGE Sample Buffer, 1 μl NativePAGE 5% G-250 Sample Additive, without (odd lanes) and with 1 μl 1 M DTT (100 mM DTT final concentration in the even lanes), and Nanopure water to 10 μl volume was run on a NativePAGE 4-16% Bis-Tris protein gel (ThermoFisher, BN1002BOX). The native gel far-red fluorescence was imaged with EX/EM = 650/690 nm with UVP ChemStudio PLUS with OptiChemi CCD Camera (Analytik Jena) (Fig. 3c). Coomassie Brilliant Blue stained the NativePAGE gel for total protein visualization (Fig. 3b). A denaturing SDS-PAGE gel was with 8 μg of purified protein per lane without (even lanes) and with 1 μl of 1 M DTT (100 mM DTT final concentration in the odd lanes), 2.5 μl 4X NuPAGE LDS Sample Buffer, and Nanopure water to 10 μl on a NuPAGE Novex 4-12% Bis-Tris gel with NuPAGE MOPS SDS running buffer. The protein ladder in Lanes 1 and 10 is Precision Plus Protein Dual Color Standards (Bio-Rad 1610374). The gel was stained with Coomassie Brilliant Blue to visualize total protein (Supplementary Fig. 8). Uncropped gels of Fig. 3b, c and Supplementary Fig. 8 are provided in the Source Data File.

### Photophysical property determination of purified FPs

The ECs and QYs were determined as described^6,11^. The Soret band absorbance at 380 nm determines the protein concentration for the EC. The 380 nm absorbance is unchanged when covalently attached to smURFP or free in solution. To confirm no change, we denature an FP with a chromophore (BV or PCB) with 1 M urea. A Jasco V-770 UV/Visible Spectrophotometer measured absorbance. The Q band EC is determined by:

EC _Max λ_ = Absorbance _Max λ_ / (Concentration * Path Length)

The QYs were determined by comparison to Cy5, where QY = 25% in PBS. The absorbance of Cy5 and FP were matched using a Jasco UV/Visible Spectrophotometer. A Horiba Fluoromax-4CP Fluorometer measured the Cy5 and FP emission excited at the same wavelength. The FP emission was integrated and divided by the integral of the Cy5 emission and then multiplied by QY = 25%.

A Digital Frequency Domain system (ChronosDFD, ISS) measured the fluorescence lifetime of smURFP+BV in PBS. Chromophores were excited with a 518 nm laser diode (ISS). Fluorescence was detected after a 561LP filter. Rose Bengal in ethanol was the reference with τ = 0.785 ns^66^ to obtain the instrument response function. The fluorescence decay was fit to a single exponential with χ^2^ = 0.98.

### CytERM-fusion plasmid construction for the OSER assay

The CytERM-mGFP plasmid was from Erik Snapp (Addgene 62237). The CytERM-smURFP and CytERM-MsmURFP plasmids were created by replacing the gene coding for mGFP with smURFP or MsmURFP, both mammalian codon-optimized. The coding sequences were PCR amplified from pcDNA3-smURFP-IRES-eGFP (Addgene 80343) or pcDNA3-MsmURFP-IRES-eGFP with Phusion HF PCR Master Mix (ThermoFisher F531L) and 5’-3’ primers GATCCACCGGTCGCCACCATGGCTAAGACTTCCGAAC and GAGTCGCGGCCGCTTTAGCTCATAGCCTTAATAATGTAATCAAAGTAG. The DNA Clean & Concentrator Kit (Zymo D4013) purified the PCR product. AgeI and NotI (NEB) cut the plasmid and PCR products. SAP (Applied Biosystems 783901000UN) dephosphorylated the digested plasmid. T4 DNA ligase (ThermoFisher 15224017) ligated digested plasmid and PCR products. One Shot OmniMAX 2 T1^R^ Chemically Competent *E. coli* (ThermoFisher C854001) were transformed and plated on prewarmed LB-agar + 50 µg/ml Kanamycin plates. The plates were incubated at 37 °C for 24 h. Single colonies were grown in liquid cultures, and plasmids were purified by QIAprep Spin Miniprep Kit (Qiagen 27106). Sanger sequencing confirmed DNA sequences.

### Mammalian cell growth, transient transfection, & stable cell lines

#### HEK293A cell transient transfection with DNA

ThermoFisher verified the HEK293A cell line (ThermoFisher R70507) identity. The cells were maintained in isolation to avoid cross-contamination. HEK cells are listed by the ICLAC as a misidentified cell line often contaminated with HeLa cells, which are not used in our laboratory. The flat HEK293A cell morphology confirmed the cellular identity of HEK293A cells before transfection and cellular imaging. HEK293A cells were grown in DMEM (Gibco 11885084) supplemented with 10% Heat Inactivated FBS (Gibco 10438026) + 1% GlutaMAX (Gibco 35050061) + 1X Penicillin-Streptomycin (MilliporeSigma P4333), which is referred to as growth medium, at 37 °C in 95% humidity + 5% CO_2_ atmosphere. Cells were randomly seeded on 35 mm, #0 or #1.5 glass-bottom poly-D-lysine-coated dishes (MatTek P35GC-0 or 1.5-10-C) and transfected at 50-80% confluency with the Lipofectamine 3000 (ThermoFisher, L3000015) using 1-2 µg of DNA.

#### OSER assay

Cells transiently expressing CytERM-mGFP were imaged at 24 or 48 h post-transfection. Cells transiently expressing CytERM-smURFP or CytERM-MsmURFP were incubated for 6 h. Fresh growth medium supplemented with 1 µM BVMe_2_ or 25 µM PCB replaced media for an additional 18 or 42 h before imaging at 24 or 48 h post-transfection.

#### MsmURFP

Cells transiently expressing smURFP/MsmURFP IRES eGFP were incubated for 45 h. We added 25 µM BV/PCB or 5 µM BVMe_2_ for 3 h.

#### Mammalian stable cell line generation

HEK293A cells were grown in growth media at 37 °C with 95% humidity + 5% CO_2_ atmosphere. Lipofectamine 3000 transfected cells with 2 µg pcDNA3-smURFP-IRES-eGFP (Addgene 80343) at 85% confluent cells in a 35 mm dish. Epifluorescence imaging (**HEK293A stable cell line epifluorescence imaging** section) verified FP expression after 48 h. Growth media supplemented with 200 µg/ml Hygromycin B selected stably expressing cells for 4 weeks. Trypsinized cells were suspended in 1X PBS (pH 7.4) + 1 mM EDTA + 0.5 mM HEPES + 1% FBS. Fluorescence-activated cell sorting (FACS, BD Biosciences Influx) selected bright single cells using filters: eGFP EX / EM = 488 (100 mW) / 530(40) nm and smURFP EX / EM = 640 (120 mW) / 670(30) nm. Epifluorescence microscopy (**HEK293A stable cell line epifluorescence imaging** section) verified eGFP and smURFP expression and proper cell growth in clonal cell lines.

### Fluorescence microscopy

Growth medium rinsed cells before imaging in Hank’s Balanced Salt Solution (HBSS, Gibco 14065-056) + 2 g/L glucose + 20 mM HEPES (pH 7.4) without phenol red and chromophore (BV, PCB, or BVMe_2_). Cell locations were randomly chosen to be representative of the entire dish.

#### OSER assay epifluorescence imaging

An Inverted Zeiss Axio Observer microscope controlled with Zeiss Zen Blue V. 2.3 software with an Axiocam503m CCD camera and a 40X magnification, 1.3 NA, immersion oil objective (EC Plan-NeoFluar DIC M27) imaged room temperature cells at 1936×1460 pixels with a resolution of 0.1135 µm/pixel. Cells were grown in #1.5 glass-bottom poly-D-lysine-coated dishes. CytERM-mGFP was imaged with a 475 nm LED with EX/EM = 475(20)/525(25) nm filters. CytERM-smURFP and CytERM-MsmURFP were imaged with a 631 nm LED with EX/EM = 630(13)/709(50) nm filters.

#### MsmURFP epifluorescence imaging

A ThermoFisher EVOS FL Auto 2 System controlled with ThermoFisher EVOS FL Auto 2 V.2.0.2094.0 software using a 40X magnification, 0.75 NA, fluorite, coverslip-corrected objective (ThermoFisher AMEP4699) acquired images at room temperature using the filters: eGFP EX/EM = 470(22)/510(42) nm, smURFP and MsmURFP EX/EM = 635(18)/692(40) nm, and Cy5.5 EX/EM = 665(40)/794(160) nm. Images were 2048 × 1536 pixels with a resolution of 3.45 µm/pixel. Cells were grown on #0 glass-bottom poly-D-lysine-coated dishes.

#### HEK293A stable cell line epifluorescence imaging

Stable cells expressing smURFP and eGFP on the same mRNA were supplemented with 1 µM BVMe_2_ overnight. A ThermoFisher EVOS FL Auto 2 System controlled with ThermoFisher EVOS FL Auto 2 V.2.0.2094.0 software imaged cells through plastic 96 well plates and #0 glass-bottom poly-D-lysine-coated dishes using the filters: eGFP EX/EM = 470(22)/510(42) nm and smURFP EX/EM = 635(18)/692(40) nm.

#### FLIM

HEK293A cells, stably expressing smURFP-IRES-eGFP and wild-type, were labeled with 1 or 10 μM BVMe_2_, respectively, for 24 h in 35 mm, #1.5 glass-bottom poly-D-lysine-coated dishes. An inverted Leica SP8 FALCON controlled by Leica Application Suite X V. 3.5.7.23225 software with a white light laser (80 MHz, 12.5 ns between pulses) tuned to 488 nm for eGFP and 642 nm for smURFP+BVMe_2_ and free BVMe_2_ with EM from 570(70) and 690(76) nm, respectively, with a 63X magnification, 1.4 NA, immersion oil objective (HC PL APO CS2). For eGFP and smURFP+BVMe_2_, 14,150,000 and 11,830,000 photons determined the fluorescence lifetime. BVMe_2_ was dimmer, and 1,000,000 photons determined the fluorescence lifetime. Fluorescence lifetimes were fit to a single exponential with χ^2^ ≤ 5.0.

### Epifluorescence image analysis and statistical comparison

ImageJ (Fiji) V. 2.3.0/1.53t^67^ analyzed epifluorescence images and included only non-saturated cells.

### OSER assay

Cells exhibiting excessive brightness and an abnormal phenotype (multiple nuclei, lobed nucleus, disorganized ER membrane, or undefined nuclear envelope) were not analyzed. Normal cells had mid-to-low fluorescence intensity and showed a reticular ER network without OSER whorl structures. Cells that showed whorls were brighter than normal cells because of the high effective concentration of the protein fusion on the ER membrane and nuclear envelope (NE). Celleste Image Analysis Software V 5.0.2.6993 (ThermoFisher) loaded and processed 16-bit, grayscale raw images using a blind 2D Deconvolution module with the default settings (Adaptive PSF, iterations = 20, noise Level = 2). Deconvolved images were background subtracted with 600×600 pixels at 9 pixels/µm (Supplementary Fig. 7a-d). The determination of the ratio = (MFI of OSER)/(MFI of NE) was performed as described in^29^ (Supplementary Fig. 7e).

### MsmURFP comparison to smURFP

In ImageJ (Fiji) V. 2.3.0/1.53t^67^, a region of interest (ROI) selected a single cell, and the same ROI measured the MFI of each cell. The ROI measured background fluorescence along the edge of the images without cells. The MFI of cells was background subtracted. Ten cells were measured in four representative images for *n* = 40 cells for each filter setting for 120 measurements. For smURFP+PCB, the fluorescent intensity was saturated at 200 ms, and the exposure was 50 ms. The MFI was assumed to be linear and multiplied by four for comparison.

### Characterization of one- & two-photon absorption spectra of smURFP

We measured the one-photon absorption spectra of smURFP+BV and BV in PBS with a Jasco V-770 UV/Visible Spectrophotometer. The smURFP absorbance was normalized at 642 nm, and BV was matched at the Soret band (370 nm) for comparison. The two-photon absorption spectrum and absolute cross-sections of smURFP were obtained as described^68^. Briefly, a LabVIEW V. 2018 program automatically tuned an Insight DeepSee (Spectra-Physics) femtosecond laser for fluorescence excitation. A PC1 (ISS) photon counting spectrofluorometer detected fluorescence intensity. The two-photon spectral scan used 694SP and 770SP fluorescence filters (Semrock FF02-694/SP-25 and FF01-770/SP-25, respectively). Absolute, two-photon cross-sections of smURFP were measured at 1,060 nm versus Rhodamine 6 G (Exciton 05901) in methanol (two-photon cross-section = 10 GM)^68^ with fluorescence registered at 665 and 1,000 nm versus LDS 798 (Exciton 07980) in chloroform (two-photon cross-section = 244 GM)^69^ with fluorescence registered at 700 nm. The 21 and 31 GM at 1060 and 1000 nm, respectively, agreed with the two-photon absorption spectrum variation between these two wavelengths.

### Single-molecule photons detected before photobleaching

smURFP+BV and TDsmURFP+BV were purified from *E. coli*. Single molecules of AlexaFluor647, Cy5, smURFP+BV, and TDsmURFP+BV were imaged as immobilized chromophores/fluorophores in polymer films. 1% (w/w) poly (vinyl alcohol) (PVA) in Nanopure water prepared polymer films. 100 pM of chromophores/fluorophores were added to PVA, and 50 µL of the solution was spin cast on argon-plasma etched glass coverslips (FisherFinest, #1.5, 22×22 mm, 12-548B). An inverted Olympus IX71 epifluorescence microscope equipped with a UPlan FLN 100x magnification, 1.3 NA, immersion oil objective, motorized stage (PILine Physik Instrumente M-687), and 638 nm solid-state laser (Blue Sky Research, FiberTec II) imaged samples as described^15^.

### Statistical Methods

KaleidaGraph V. 5.0.4 (Synergy Software) calculated statistics in Fig. 3d and Supplementary Fig. 7e, and plotted graphs in Figs. 4a, b, and 5, Supplementary Figs. 3b, c, and 7e. KaleidaGraph calculated *p* values with a one-way ANOVA with a significance (α) of 0.05 with a *post hoc* test of Tukey honestly significant difference (HSD) when comparing two ratios. Error bar specification and sample size (*n*) are listed with each experiment.

### Reporting summary

Further information on research design is available in the Nature Portfolio Reporting Summary linked to this article.

## Supplementary information

Supplementary Information

Description of additional supplementary files

Supplementary Movie 1

Supplementary Movie 2

Supplementary Movie 3

Supplementary Movie 4

Supplementary Movie 5

Supplementary Movie 6

Reporting Summary

## Data Availability

The smURFP crystal structure is available at the Protein Data Bank under accession code 7UQA. The following crystal structures were used in this study, Protein Data Bank accession codes 4RMP^39^, 6FZN^28^, 6FZO^28^, and 4PO5^47^. GenBank/EMBL/DDBJ ascension codes are KX449134 and KX449135 for smURFP and TDsmURFP, respectively. Plasmid DNA for smURFP and TDsmURFP for bacterial and mammalian expression is available from Addgene (80341, 80342, 80343, 80344) [https://www.addgene.org/Erik_Rodriguez/]. The source data for Figs. 3d, 4a-b, 5, and Supplementary Figs. 3a-c, 7e-f are provided in the Excel Source Data file with this paper. Additionally, the smURFP structures with one and two BV (Supplementary Structures 1, 2) are provided in the Source Data file. Source data are provided with this paper.

## Source data

Source Data
