# Supplementary material for: Structural and photophysical characterization of the small ultra-red fluorescent protein
Source: Nat Commun. 2023 Jul 12;14:4155. doi: 10.1038/s41467-023-39776-9 (PMC10338489; doi:10.1038/s41467-023-39776-9)
Supplement: Supplementary file 1 — Supplementary Information [file 41467_2023_39776_MOESM1_ESM.pdf]

Supplementary Information for

Structural and photophysical characterization of the small ultra-red fluorescent protein

Authors

Atanu Maiti[1], Cosmo Z. Buffalo[2], Saumya Saurabh[3,4], Felipe Montecinos-Franjola[5], Justin S. Hachey[5], William J. Conlon[5], Geraldine N. Tran[6], Bakar Hassan[7], Kylie J. Walters[7], Mikhail Drobizhev[8], W. E. Moerner[3], Partho Ghosh[9], Hiroshi Matsuo[1], Roger Y. Tsien[9,10], John Y. Lin[11], and Erik A. Rodriguez[5]*.

This PDF file includes **Page #**

Supplementary Figures

Suppl. Fig. 1 smURFP protein characterization and crystals for structure determination……...................2
Suppl. Fig. 2 A region of the smURFP structure with electron density map………………………...….3
Suppl. Fig. 3 The free energy of BV binding and acid denaturation of smURFP+BV……………………4
Suppl. Figs. 4-6 Mutation locations of the smURFP directed evolution from Reference 3……………..5-7
Suppl. Fig. 7 The OSER assay detected oligomerization of smURFP……………………...…………..8
Suppl. Fig. 8 Denaturing gel confirmed monomeric smURFP variants are the correct size.…...……........9
Suppl. Fig. 9 Protein sequence alignment of smURFP and *Phormidium* sp. A09DM allophycocyanin…10
Suppl. Fig. 10 Alignment of the smURFP homodimer to the α- and β-allophycocyanin dimer…………11
Suppl. Fig. 11 Comparison of the smURFP asymmetric crystal unit hexamer to the allophycocyanin hexamer……………………………………………………………………………………….…12
Suppl. Fig. 12 Allophycocyanin dimer interactions…………………………………….…………..13
Suppl. Fig. 13 Comparison of the smURFP BV pocket to the α-allophycocyanin PCB pocket…...…….14
Suppl. Fig. 14 Protein sequence alignment of smURFP, smURFP Y56F, and smURFP Y56R…………15
Suppl. Fig. 15 Comparison of smURFP to mutant crystal structures……………………………………16

Supplementary Tables

Suppl. Table 1 Data collection and refinement statistics of the smURFP crystal structure……..………..17
Suppl. Table 2 Summary of the smURFP directed evolution with mutation locations & photophysical properties…………………………………………………………………………………………18
Suppl. Table 3 smURFP, smURFP Y56R, and smURFP Y56F chromophore pocket and total protein volumes. ………………………………………………………………………19

Supplementary References ………………………………….……………………………………..20

Other Supplementary Information for this manuscript includes the following:

Supplementary Movies 1-6, Supplementary Structures 1,2, and Source Data File.

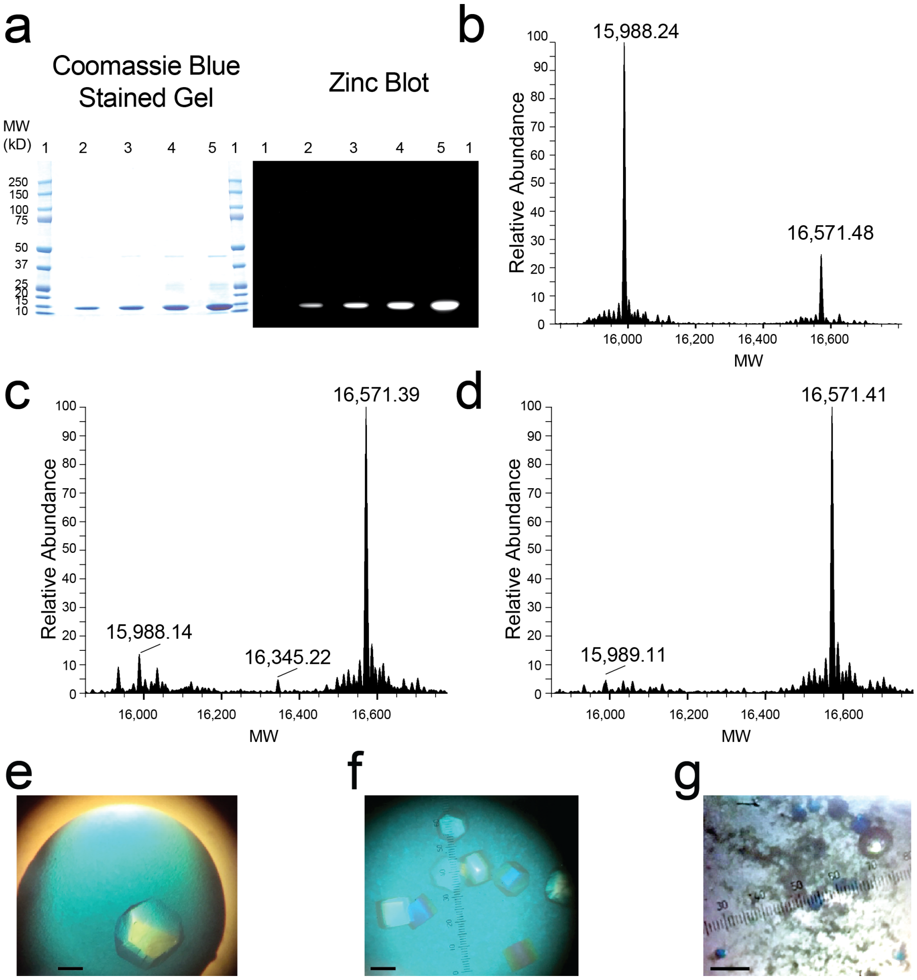

Supplementary Fig. 1 smURFP protein characterization and crystals for structure determination. a SDS-PAGE of 1.1, 2.2, 4.4, and 8.8 μg of smURFP+BV (0.02% Arabinose in LB) was loaded in lanes 2, 3, 4, and 5, respectively. Left is total protein stained with Coomassie Brilliant Blue. Right is a Zinc blot, where Zinc binds to BV on denatured protein to produce a rigid BV helix for fluorescence imaging. $n = 1$ gel of protein in **b**. **b** Mass spectrometry of smURFP expressed in *E. coli* with *0.02% Arabinose* induction for higher protein expression. Mass spectrometry shows 20% of smURFP contains BV covalently attached at 16,571.48 D (Predicted: 16,572.07 D). smURFP lacking BV is found at 15,988.24 (Predicted: 15,989.42 D) and is 80% of the purified protein. **c** Mass spectrometry of smURFP expressed in *E. coli* with *0.002% Arabinose* induction for lower protein expression. Mass spectrometry shows 87% of smURFP contains BV covalently attached at 16,571.39 D (Predicted: 16,572.07 D). Protein lacking BV is found at 15,988.14 (Predicted: 15,989.42 D) and is 13% of purified protein. **d** Purified smURFP+BV from (**c**) with 10-fold molar excess BV added *in vitro* to saturate smURFP sites. Mass spectrometry shows ≥97% of smURFP contains BV covalently attached at 16,571.41 D (Predicted: 16,572.07 D). Protein lacking BV is found at 15,989.11 (Predicted: 15,989.42 D) and is ≤3% of total relative protein. The saturated smURFP+BV was crystallized in **g**. We obtained each mass spectra from a single injection ($n = 1$ protein purification) of purified FP (>30 μM), and each sample was repeated with three separate injections. **e** & **f** smURFP crystals formed from protein in **b** and used to determine the structure. **g** Representative image of crystals formed from saturated smURFP from **d**. The images in **e-g** are representative of $n = 30$ hanging drops. The scale bars are 100 μm.

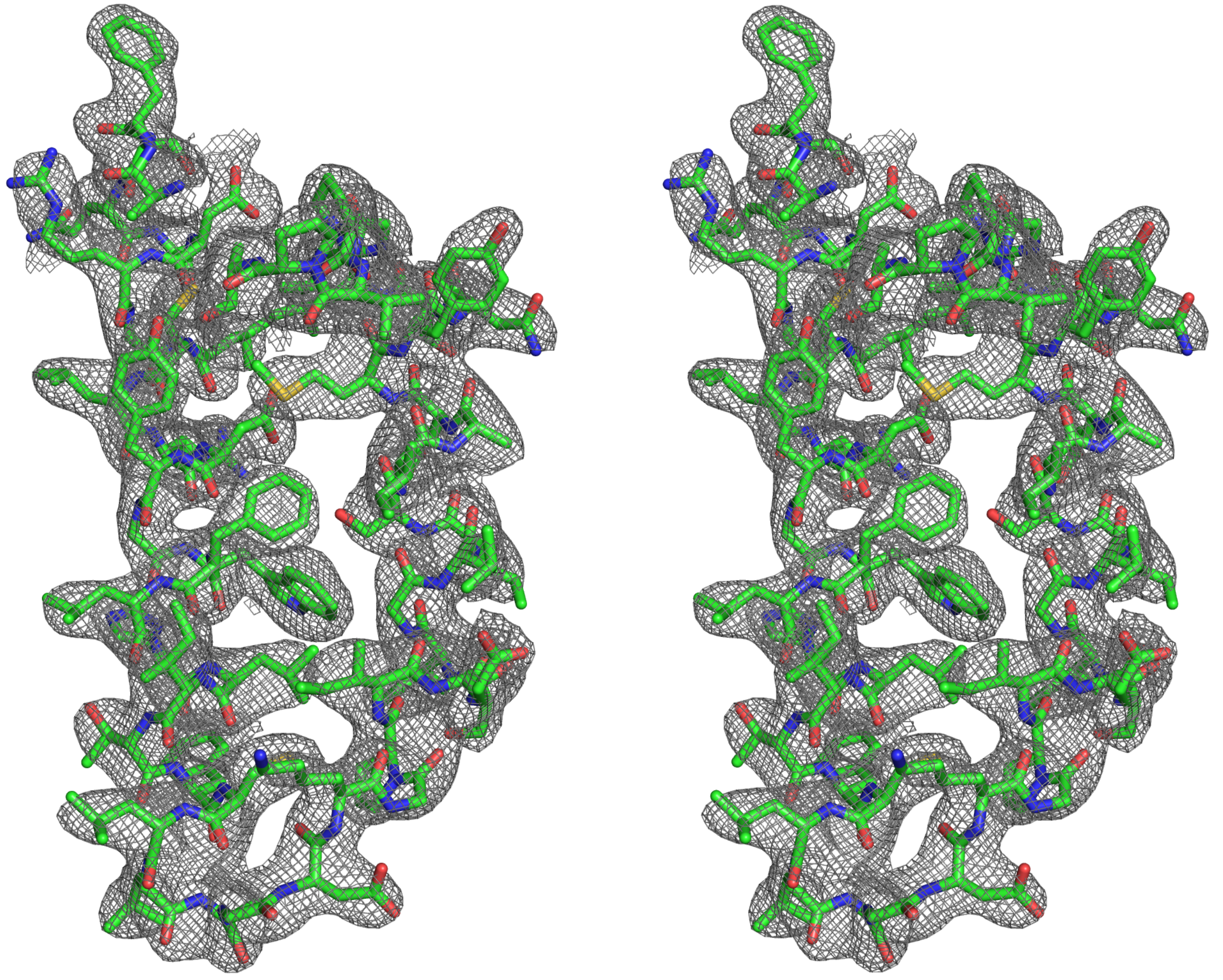

Supplementary Fig. 2 A region of the smURFP structure with electron density map. A stereo image of a portion of the electron density map (contoured at 1σ) of the smURFP chain A from Ala43 to Ala96. We determined the smURFP structure at 2.8 Å resolution.

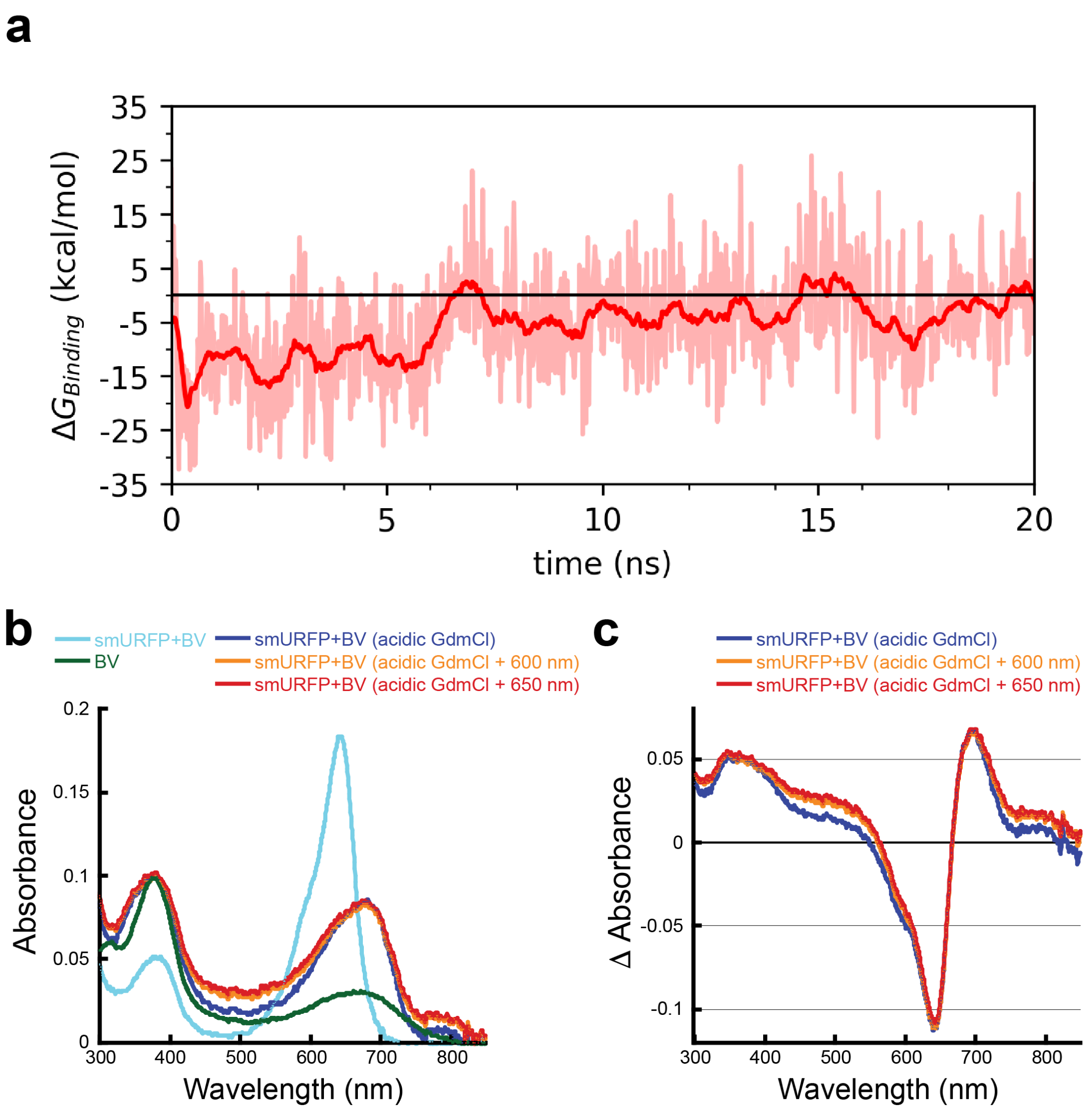

Supplementary Fig. 3 The free energy of BV binding and acid denaturation of smURFP+BV. a The smURFP with one BV complex free energy of binding ($\Delta G_{Binding}$) was calculated at each trajectory frame by the molecular mechanics/generalized Born surface area (MM/GBSA) method[1]. The simulation equilibrates over 20 nanoseconds with an average $\Delta G_{Binding}$ = -5.7±0.3 kcal/mol, where the error is the standard error of the mean (SEM) and n = 1,002 frames of MD simulation. Extracted energies from each frame are pink and the moving window average energy is the solid red line. **b** Absorption spectra of smURFP+BV (cyan) and BV (green). smURFP+BV was denatured in acidic guanidinium chloride (acidic GdmCl) without and with 600 or 650 nm illumination at room temperature for 5 min as described[2]. Each sample was repeated three times with a single representative spectrum shown. **c** Difference spectra of listed conditions relative to the folded smURFP+BV. Denaturation resulted in a red-shifted absorbance at the Q band and increased absorbance at the Soret band. The difference spectra indicate that smURFP+BV is in the 15*E* conformation. Acidic GdmCl denaturation is sufficient to convert the 15*E* BV to the 15*Z* conformation, and light illumination was not required to convert BV to the thermally stable 15*Z* form.

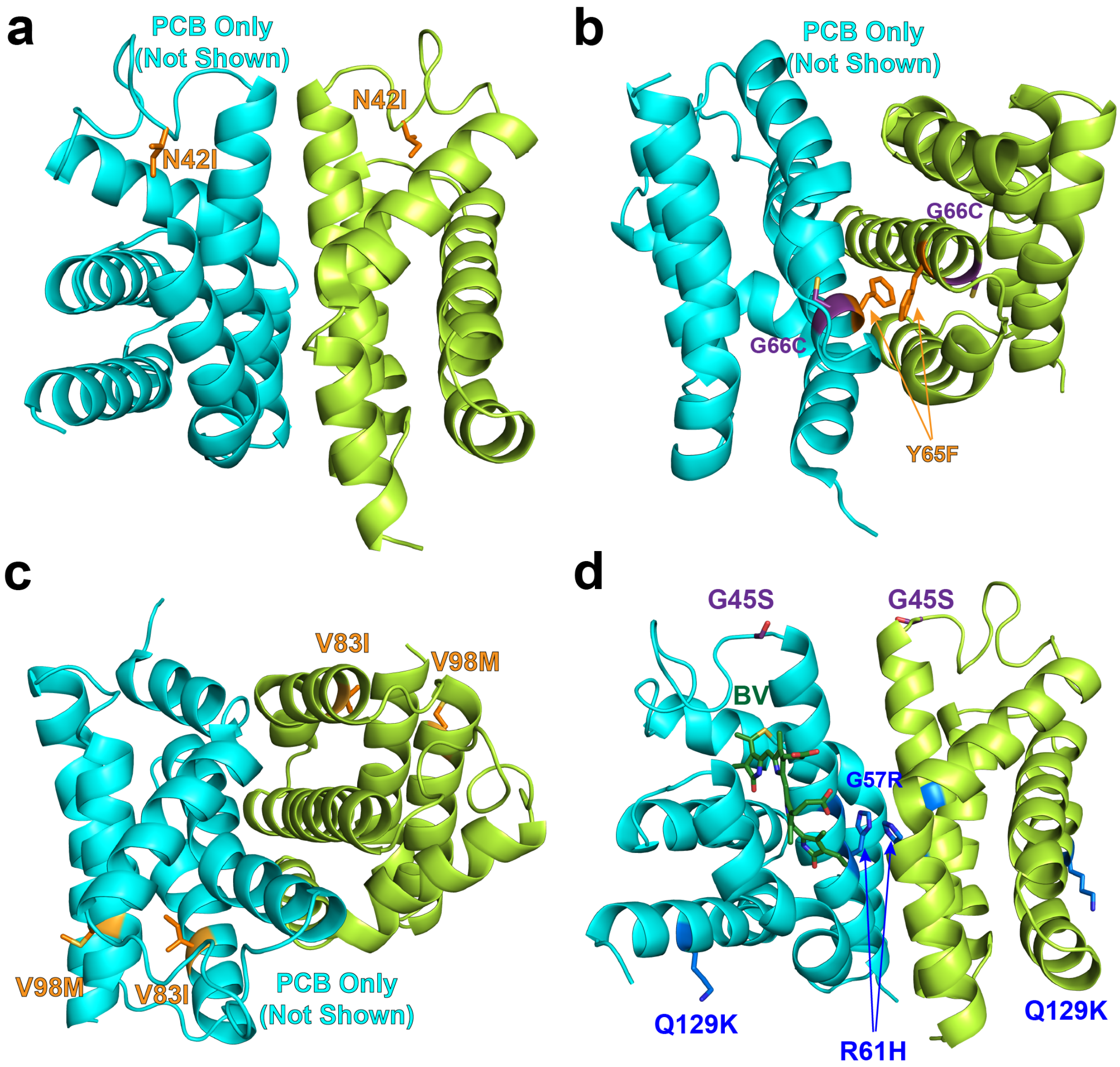

Supplementary Fig. 4 Rounds 1-4 mutation location of the smURFP directed evolution from Reference 3[3]. Supplementary Table 2 summarizes the directed evolution and photophysical properties. **a** Round 1 selected PCB incorporation. A single mutation, N42I, allowed for the incorporation of PCB. The mutation did not incorporate BV. **b** Round 2 selected enhanced fluorescence with PCB. The T65F and G66C mutations are at the dimeric interface and enhanced the fluorescence 30-fold in bacteria. **c** Round 3 selected PCB incorporation. V83I is in the chromophore pocket, while the V98M mutation is on the exterior of the dimer. The fluorescence is 50-fold brighter than R1+PCB. R1+PCB, R2-1+PCB, and R3-2+PCB did not incorporate BV. **d** Round 4 selected incorporation of BV. Four additional mutations allowed BV incorporation, G45S, G57R, R61H, and Q129K. G45S and Q129K are on the protein exterior. G57R is at the dimeric interface. R61H is in the chromophore pocket of the adjacent protomer (Supplementary Fig. 13). BV incorporation required the N42I, G45S, G57R, R61H, Q129K, Y65F, G66C, V83I, and V98M mutations. BV is colored dark green.

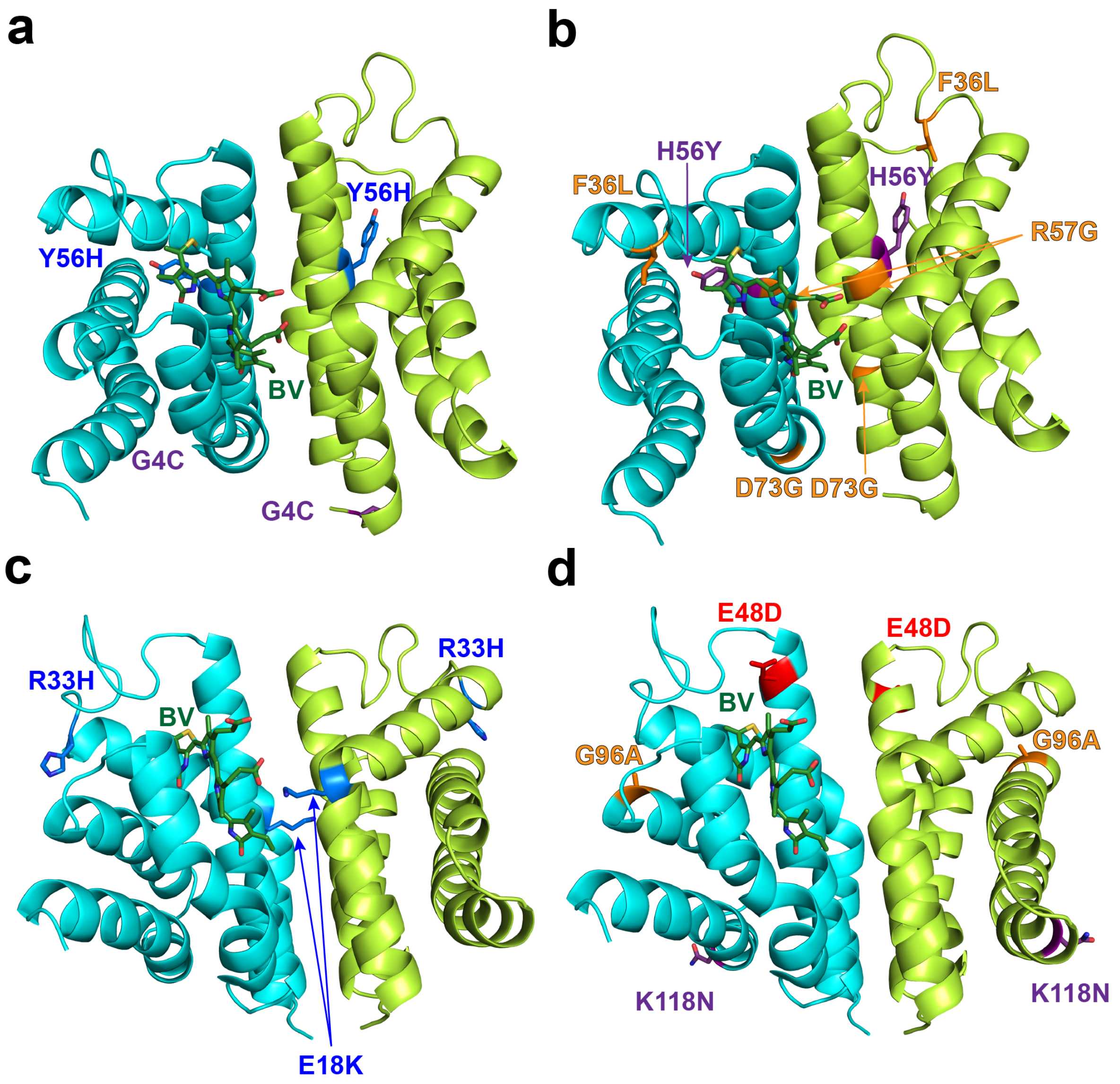

Supplementary Fig. 5 Rounds 5-8 mutation location of the smURFP directed evolution from Reference 3[3]. Supplementary Table 2 summarizes the directed evolution and photophysical properties. **a** Round 5 selected BV incorporation. G4C was on the exterior of the fluorescent protein, while the Y56H mutation resulted in red-shifted fluorescence and significantly decreased quantum yield (Supplementary Table 2). **b** By changing the filters, we selected blueshifted fluorescence to avoid further decreased photophysical properties in Round 6. H56Y and R57G mutations reverted to the original sequence. F36L is in the chromophore pocket and blueshifted fluorescence. D73G is at the start of an α-helix and stabilized helix initiation next to P74. **c** In Round 7, we continued to blueshift fluorescence and selected E18K, R33H, and M131I mutations on the exterior of the fluorescent protein. E18K forms a salt bridge with D55 on the second protomer to stabilize the dimer. R33H forms an intrasubunit salt bridge with E99. **d** Round 8 selected enhanced expression in bacteria. M131I reverted to the original M131. G96A rigidized BV in the chromophore pocket. E48D is on the exterior and helps shape the chromophore pocket. K118N is on the protein exterior and stabilizes expression. R8-8+BV was 310-fold brighter than R1+PCB and had the largest EC of 260,000 M^{-1} cm^{-1}. BV is colored dark green.

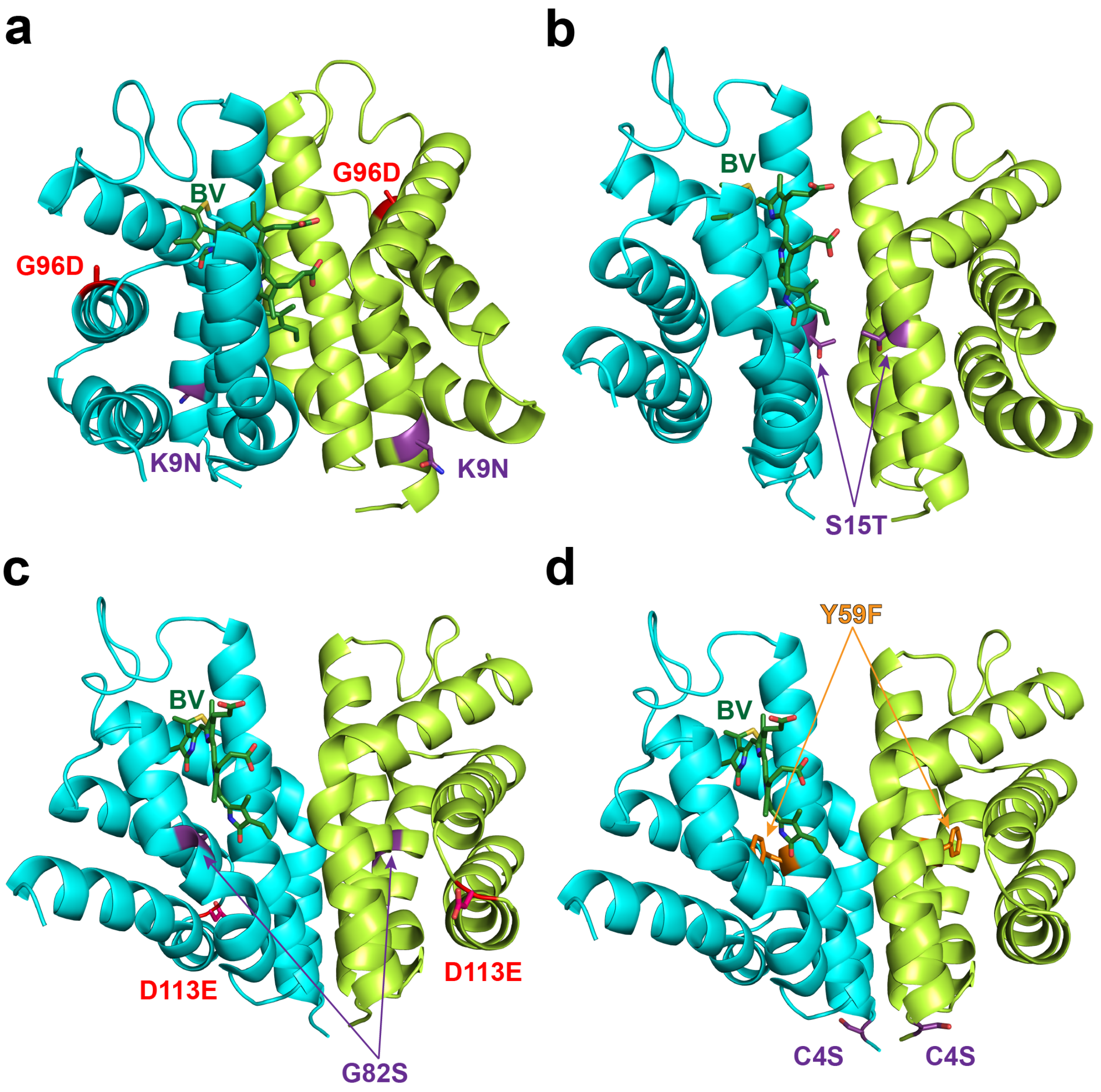

Supplementary Fig. 6 Rounds 8-12 mutation location of the smURFP directed evolution from Reference 3[3]. Supplementary Table 2 summarizes the directed evolution and photophysical properties. **a** A second clone was selected in Round 8. R8-9 contained a G96D mutation, but R8-8 G96A dominated in subsequent rounds. K9N mutation was on the exterior to enhance expression. The R8-9+BV was 295-fold brighter than R1+PCB in bacteria. **b** Round 9 did not produce an improved fluorescent protein. The 10th round selected the S15T mutation in the chromophore pocket of the adjacent protomer. The mutation added a methyl group to rigidize the chromophore and enhanced the QY by 25% relative to R8-9. R10-10 was 380-fold brighter than R1+PCB in bacteria. **c** Round 11 selected G82S mutation in the chromophore pocket. The D113E mutation was on the protein exterior. R11-2 lacked photophysical improvement relative to R10-10 but was 418-fold brighter than R1+PCB in bacteria due to enhanced expression. **d** Round 12 identified smURFP with the Y59F mutation in the chromophore pocket. Y59F increased the QY by 50% for the highest QY of 18%. The E113D mutation reverted to the original sequence E113. We mutated C4S to avoid disulfide bond formation. smURFP was 650-fold brighter than R1+PCB in bacteria. BV is colored dark green.

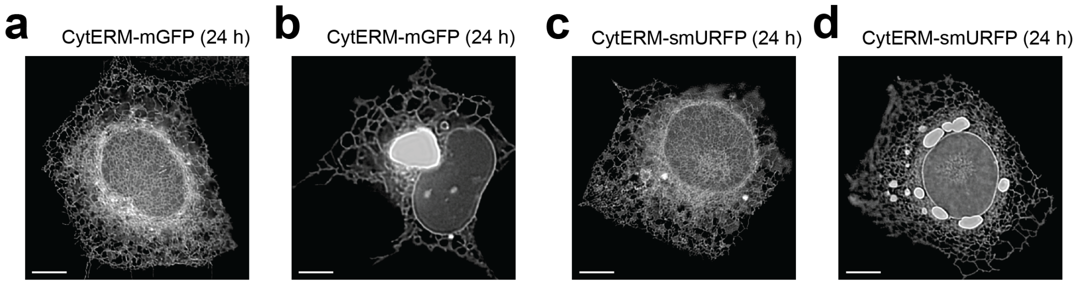

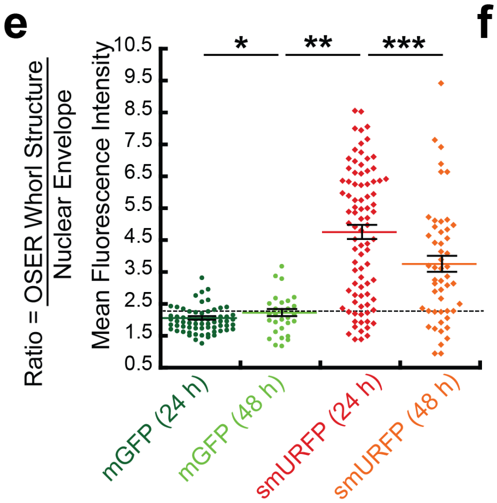

f

	mGFP (24 h)	mGFP (48 h)	smURFP (24 h)	smURFP (48 h)
Total Cells Measured (*n*)	65	29	87	51
Number of OSER Structures	46	15	236	109
Number of Cells Positive for OSER Structures	11	6	59	38
% Normal Cells	83	79	32	25
Number of Normal Cells	54	23	28	13
Average Ratio ± SEM	2.0 ± 0.05	2.2 ± 0.1	4.7 ± 0.2	3.7 ± 0.3

Supplementary Fig. 7 The organized smooth endoplasmic reticulum (OSER) assay detected oligomerization of smURFP. The assay fuses the fluorescent protein to the first 29 amino acids of the N-terminus transmembrane domain of cytochrome p450 (CytERM) for expression on the cytoplasmic face of the endoplasmic reticulum. The assay detects whether the FP fusions on opposing ER membranes associate with each other and restructure the normal ER tubular network, forming OSER whorl structures. **a-d** Epifluorescence images of normal (**a** & **c**) and abnormal (**b** & **d**) ER networks in HEK293A cells after 24 h transfection. The sample size (*n*) for each condition is in the row entitled Total Cells Measured (*n*) in **f**. The scale bars are 10 μm. **a** CytERM-mGFP with normal, reticular ER network and (**b**) abnormal ER network with an OSER whorl structure. **c** CytERM-smURFP with normal, reticular ER network and (**d**) abnormal ER network with many OSER whorl structures. **e** Quantification of the OSER assay by measuring the mean fluorescence intensity of the OSER whorl structures and dividing it by the nuclear envelope intensity to determine the ratio. The ratio is plotted for each cell at 24 and 48 h after transfection, and the mean ratio is the column width, horizontal bar. The black dotted line at 2.3 represents the monomeric FP threshold. The sample size (*n*) for each condition is in the row entitled Total Cells Measured (*n*) in **f**. * is $p = 0.11$, ** is $p < 0.0001$, and *** is $p = 0.0052$ calculated with a one-way ANOVA with significance (α) of 0.05 and error bars are standard error of the mean (SEM). There is a significant difference between 24 and 48 h for smURFP but no statistical difference for mGFP. **f** Summary table of OSER assay results at 24 and 48 h post-transfection. Normal cells displayed reticular ER without OSER whorl structures (**a** & **c**). CytERM-mGFP shows 83% and 79% normal cells after 24 and 48 h, respectively. CytERM-smURFP shows 32% and 25% normal cells after 24 and 48 h, respectively. The OSER whorl structure / Nuclear Envelope Ratio for CytERM-smURFP changes significantly from 4.8 ± 0.2 after 24 h to 3.7 ± 0.3 after 48 h ($p = 0.0052$). The lower ratio is caused by an increase in nuclear envelope mean fluorescence intensity (ratio denominator) relative to the increase in ER structure mean fluorescence intensity (ratio numerator).

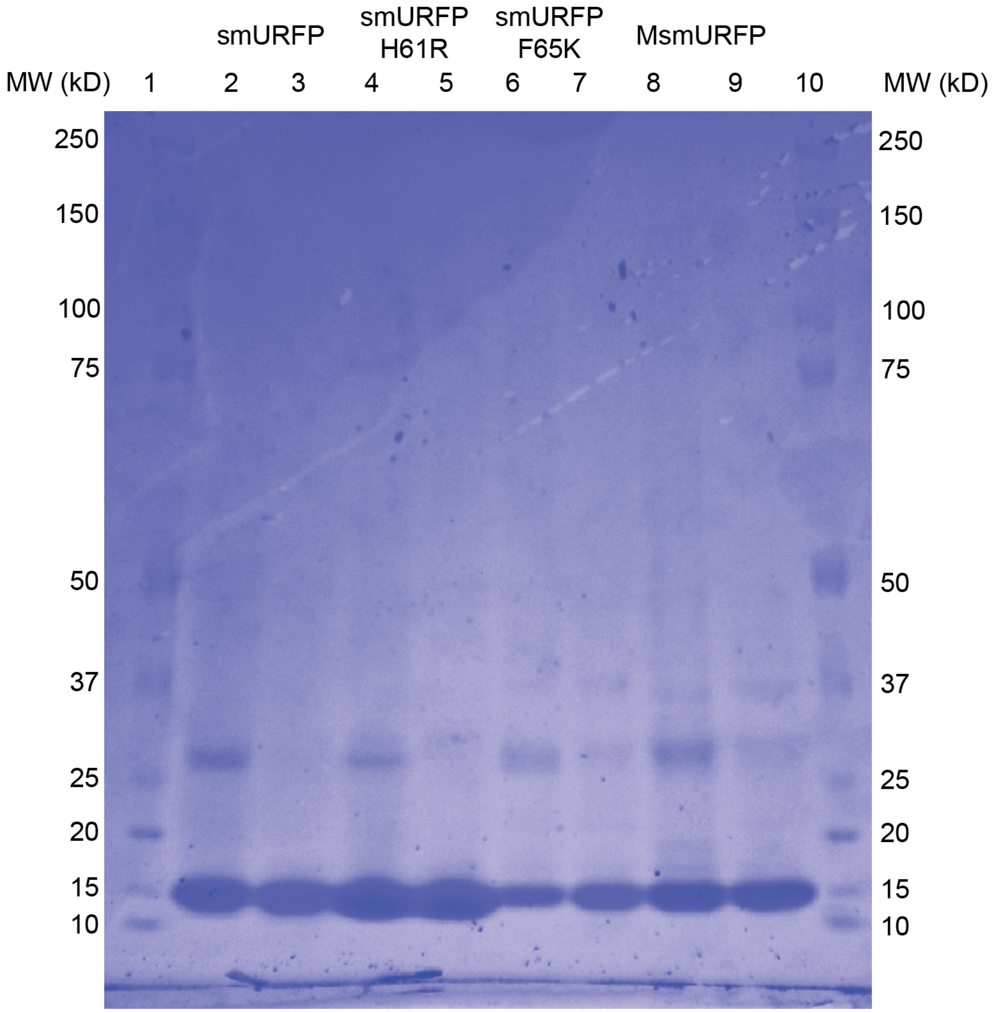

Supplementary Fig. 8 Denaturing gel confirmed monomeric smURFP variants are the correct size. The denatured proteins are the expected monomer mass of ~16 kD (smURFP-His$_6$ = 15,990 D, smURFP H61R-His$_6$ = 16,009, smURFP F65K-His$_6$ = 15,971, and MsmURFP-His$_6$ = 16,005 D). smURFP F65K was a doublet on the NativePAGE gel (Fig. 3b). Proteins of different lengths did not cause the doublet band (Lanes 6 & 7). Denatured protein formed disulfide bonds in an oxidizing environment (even lanes), and DTT addition reduced the disulfide bonds (odd lanes). *n* = 1 gel of two independent protein purifications.

```
                                                          1 [         .         .         .         .         . 50
                          1 [         .         .         .         50        .         .         .         . 80
smURFP                    ------------------------------MKTSEQRVNIATLLTENKKKIVDKASQDLWRRHPDLIAPGGIAFSQRDRA
Phor a-allophycocyanin    -MSIVTKSIVNADAEARYLSPGELDRIKGFVTSGERRLRIAQVLTESRERIVKQAGDQLFQKRPDVVSPGGNAYGEEMTA
Phor b-allophycocyanin    MQDAITAVINSSDVQGKYLDGSAMEKLKAYFQTGELRVRAATTISANAAEIVKDAVAKSL-LYSDITRPGGMMYTTRRYA
     Consensus Sequence   ..............................hpouE.RlphAphlotsttcIVcpAstp.h.hhsDlhtPGG.haspc.hA

                         51         .         70        .         .        100        .         .         . 130
                         81         .        100        .         .         .         .         :         . 160
smURFP.                   LCLRDYGWFLHLITFCLLAGDKGPIESIGLISIREMYNSLGVPVPAMMESIRCLKEASLSLLDEEDANETAPYFDYIIKA
Phor a-allophycocyanin    TCLRDMDYYLRLITYGVVAGDVTPIEEIGLVGVREMYNSLGTPIPAVAEAVRCMKSVASSLLSGEDAAEAASYFDYVVGA
Phor b-allophycocyanin    ACIRDLDYYLRYSTYAMLAGDPSILDERVLNGLKETYNSLGVPVGATVQAIQAMKEVTATLVGADAGKEMGVYFDYICSG
     Consensus Sequence   hClRDhsaaL+h.TashlAGD.s.l-phsL.ul+EhYNSLGsPlsAhhpulpshKpss.oLlst-sutEhusYFDYlhtu

                        131  ] 132
                        161  ] 162      % Sequence Identity to smURFP
smURFP                    M-                      100%
Phor a-allophycocyanin    MQ                       40%
Phor b-allophycocyanin    LS                       28%
     Consensus Sequence   h.

Consensus Sequence Legend:
o = alcohol, (S, T)
l = aliphatic (I, L, V)
a = aromatic (F, H, W, Y)
c = charged (D, E, H, K, R)
h = hydrophobic (A, C, F, G, H, I, K, L, M, R, T, V, W, Y)
- = negative (D, E)
p = polar (C, D, E, H, K, N, Q, R, S, T)
+ = positive (H, K, R)
s = small (A, C, D, G, N, P, S, T, V)
u = tiny (A, G, S)
t = turn like (A, C, D, E, G, H, K, N, Q, R, S, T)
. = no consensus
```

Supplementary Fig. 9 Protein sequence alignment of smURFP and *Phormidium* sp. A09DM allophycocyanin. *Phor* a-allophycocyanin and *Phor* b-allophycocyanin are the α- and β-subunits, respectively. We evolved smURFP from the marine cyanobacterium *Trichodesmium erythraeum* α-allophycocyanin[3], which shares a 40% sequence identity with the marine cyanobacterium *Phormidium* sp. A09DM α-allophycocyanin. As expected, the *Phormidium* sp. A09DM β-allophycocyanin shows less homology with 28% sequence identity to smURFP. smURFP lacks the first 29 amino acids. The numbers above the sequences are for smURFP, while the numbers below are the *Phormidium* allophycocyanin. C52 (yellow) covalently attaches BV in smURFP and PCB in the three proteins. Conserved amino acids are highlighted with the following classification: positive, negative, hydrophobic, and polar. The smURFP sequence is from [3], and the *Phormidium* sp. A09DM allophycocyanin sequences are from [4].

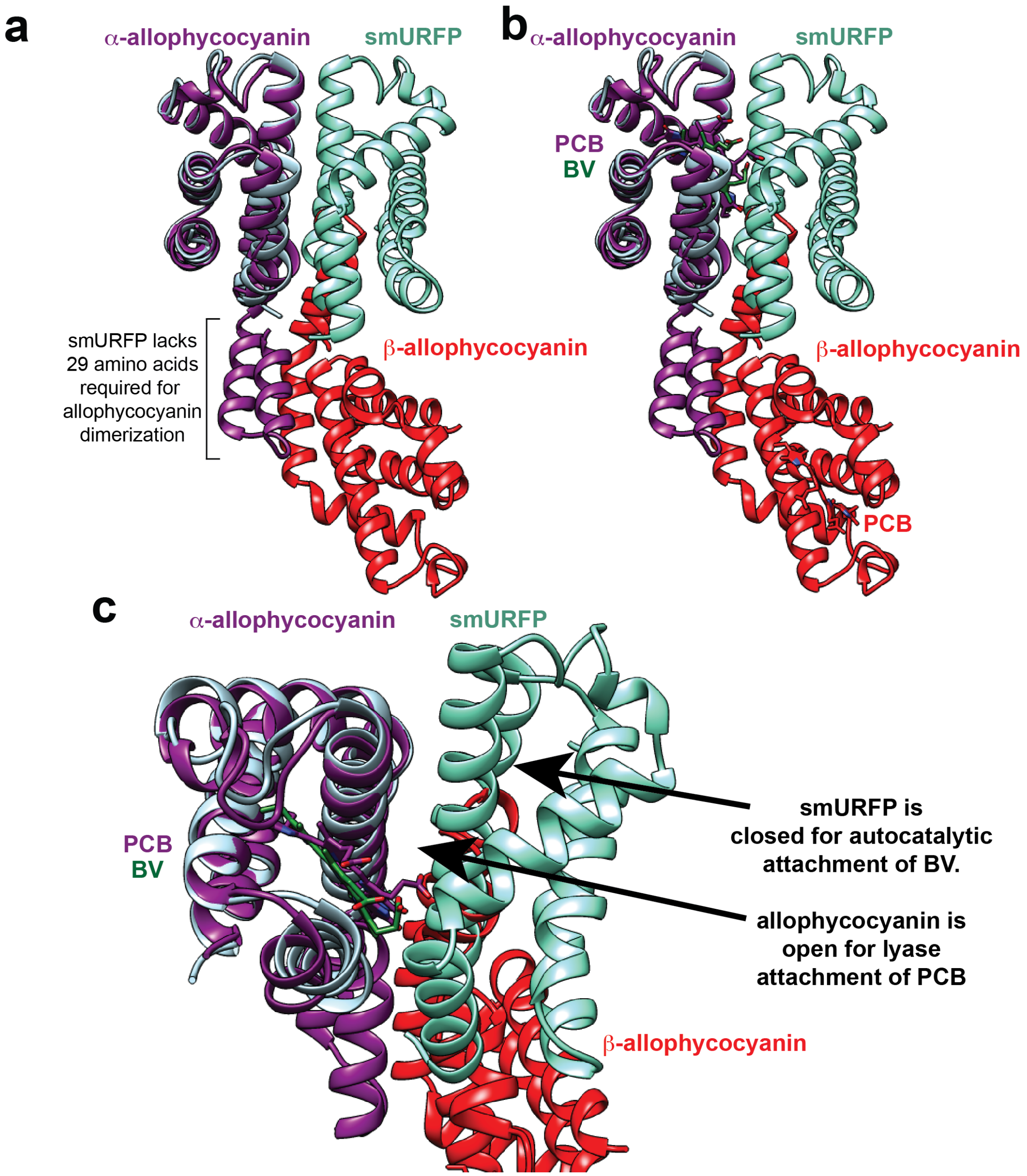

Supplementary Fig. 10 Alignment of the smURFP homodimer to the α- and β-allophycocyanin dimer. **a** The smURFP homodimer (cyan, PDB ID: 7UQA [https://doi.org/10.2210/pdb7UQA/pdb]) was aligned to the α- and β-allophycocyanin dimer (PDB ID: 4RMP [https://doi.org/10.2210/pdb4RMP/pdb])[4]. The α-allophycocyanin is magenta, and the β-allophycocyanin is red. smURFP lacks 29 amino acids common to the N-terminus of allophycocyanin proteins (Supplementary Fig. 9). The directed evolution created a new dimer interface for the covalent attachment of BV. **b** smURFP incorporated BV (dark green). A lyase covalently attached PCB (dark magenta and red) to α- and β-allophycocyanin, respectively. **c** BV attachment to smURFP includes amino acids from both protomers, and the pocket is closed for incorporation without a lyase. PCB attachment requires lyase binding, and the pocket is open.

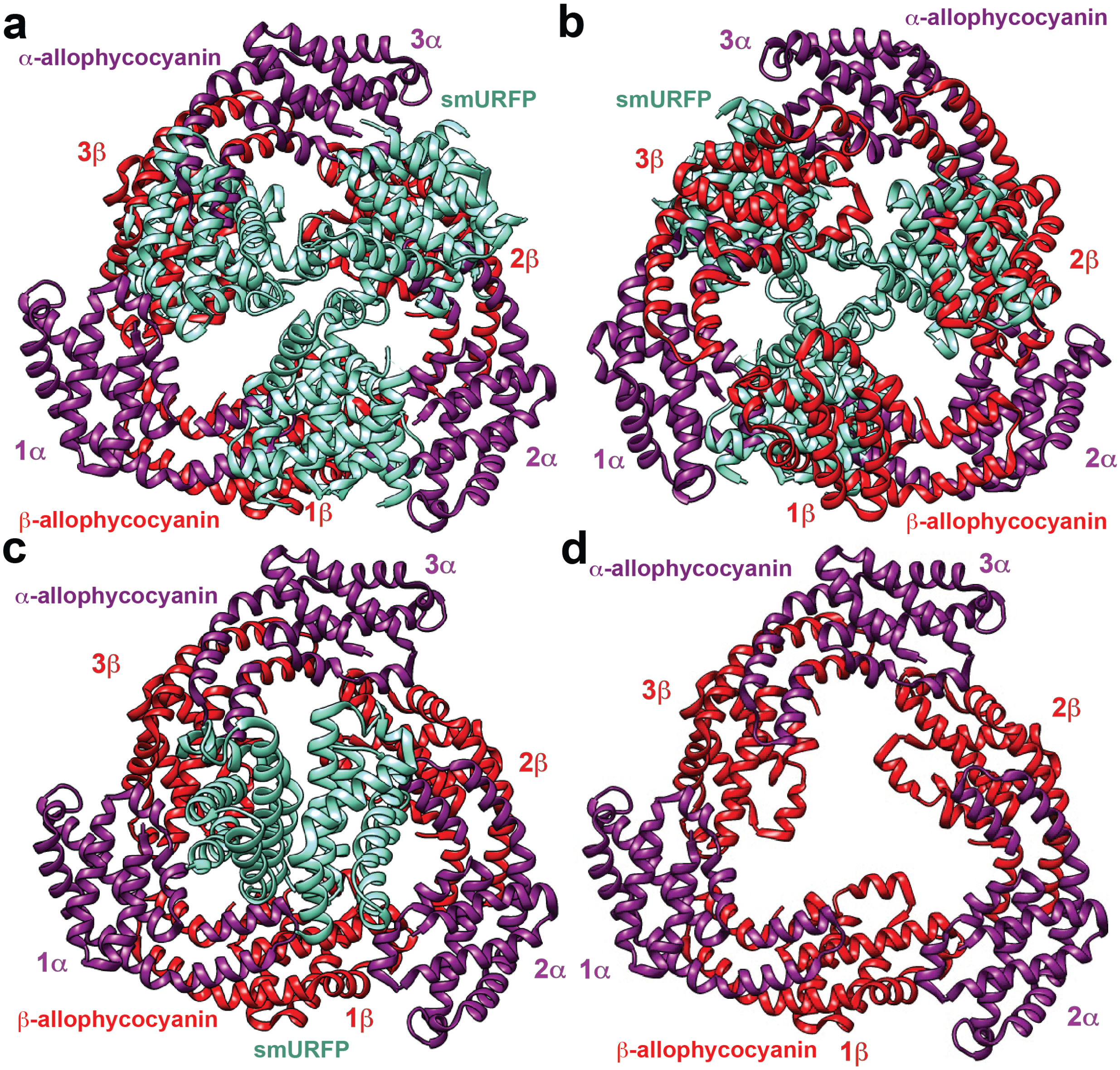

Supplementary Fig. 11 Comparison of the smURFP asymmetric crystal unit hexamer to the allophycocyanin hexamer. **a** Our smURFP hexamer (cyan, PDB ID: 7UQA [https://doi.org/10.2210/pdb7UQA/pdb]) is shown in front of the allophycocyanin hexamer from the marine cyanobacterium *Phormidium* sp. A09DM (PDB ID: 4RMP [https://doi.org/10.2210/pdb4RMP/pdb])[4]. The α-allophycocyanin is magenta, and the β-allophycocyanin is red. The molecular weight of the allophycocyanin hexamer is 118 kD. The smURFP hexamer molecular weight is 3 X 32 kD = 92 kD. **b** The *Phormidium* sp. A09DM allophycocyanin hexamer in front of the smURFP hexamer. **c** The fluorescent smURFP homodimer (32 kD) is shown before the allophycocyanin hexamer (118 kD). smURFP lacks 29 amino acids common to the N-terminus of α-allophycocyanin proteins (Supplementary Fig. 9). The smURFP directed evolution created a new dimer interface to attach BV. **d** The allophycocyanin hexamer (PDB ID: 4RMP [https://doi.org/10.2210/pdb4RMP/pdb])[4].

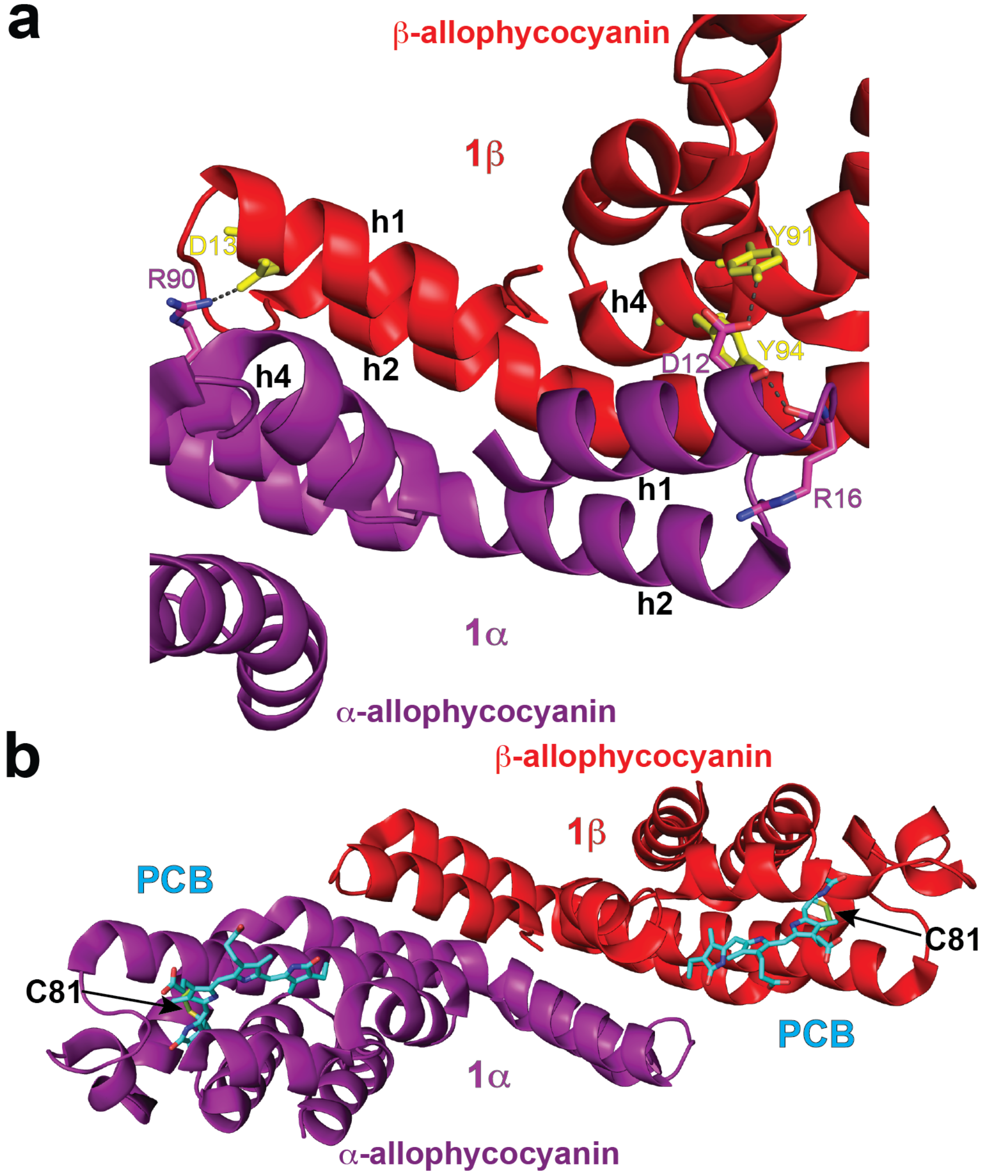

Supplementary Fig. 12 Allophycocyanin dimer interactions. a The allophycocyanin dimeric interface is formed by helices 1, 2, and 4 (h1, h2, and h4) through residue hydrogen bonds. We show important hydrogen bonding residues. **b** The PCB chromophore pockets of the allophycocyanin dimer. C81 covalently attaches PCB in a reaction catalyzed by an external lyase. PCB is cyan, while nitrogen, oxygen, and sulfur are colored blue, red, and yellow, respectively. The α-allophycocyanin structure is PDB ID: 4RMP [https://doi.org/10.2210/pdb4RMP/pdb][4].

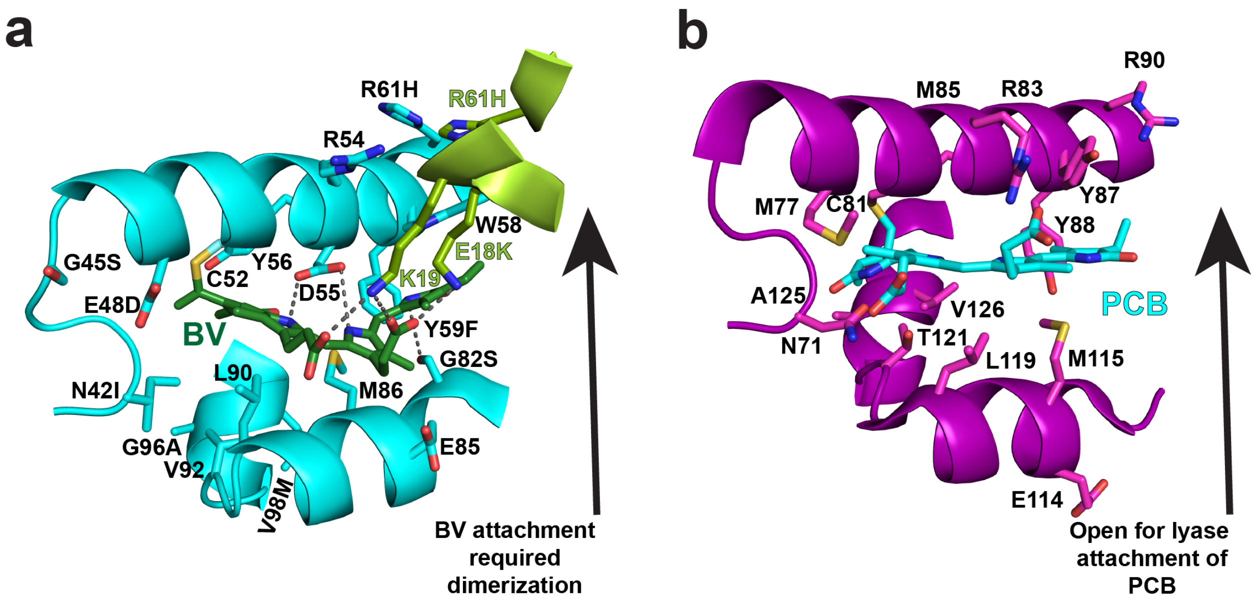

Supplementary Fig. 13 Comparison of the smURFP BV pocket to the α-allophycocyanin PCB pocket. a Our smURFP structure (PDB ID: 7UQA [https://doi.org/10.2210/pdb7UQA/pdb]) with BV covalently attached (Supplementary Structure 1). We show amino acids ≤5 Å from the BV and selected mutations from the directed evolution. The smURFP protomers are cyan and light green. BV incorporation requires dimerization for incorporation. BV is dark green, while nitrogen, oxygen, and sulfur are colored blue, red, and yellow, respectively. **b** The α-allophycocyanin structure with PCB covalently attached (PDB ID: 4RMP [https://doi.org/10.2210/pdb4RMP/pdb])[4]. We show amino acids ≤5 Å from the PCB. A lyase catalyzes PCB incorporation, and the pocket is open. PCB is cyan, while nitrogen, oxygen, and sulfur are colored blue, red, and yellow, respectively.

```
                                                                               52  56
smURFP         MKTSEQRVNIATLLTENKKKIVDKASQDLWRRHPDLIAPGGIAFSQRDRALCLRDYGWFL      60
smURFP Y56F    MKTSEQRVNIATLLTENKKKIVDKASQDLWRRHPDLIAPGGIAFSQRDRALCLRDFGWFL      60
smURFP Y56R    MKTSEQRVNIATLLTENKKKIVDKASQDLWRRHPDLIAPGGIAFSQRDRALCLRDRGWFL      60
               *******************************************************X****

smURFP         HLITFCLLAGDKGPIESIGLISIREMYNSLGVPVPAMMESIRCLKEASLSLLDEEDANET      120
smURFP Y56F    HLITFCLLAGDKGPIESIGLISIREMYNSLGVPVPAMMESIRCLKEASLSLLDEEDANET      120
smURFP Y56R    HLITFCLLAGDKGPIESIGLISIREMYNSLGVPVPAMMESIRCLKEASLSLLDEEDANET      120
               ************************************************************

smURFP         APYFDYIIKAMSEFHHHHHH      140
smURFP Y56F    APYFDYIIKAMS--HHHHHH      138
smURFP Y56R    APYFDYIIKAMS--HHHHHH      138
               ************__******
```

Supplementary Fig. 14 Protein sequence alignment of smURFP, smURFP Y56F, and smURFP Y56R. C52 (yellow) covalently attaches BV. Position 56 (cyan) shows mutations. The smURFP sequence is from [3], and the smURFP Y56F and smURFP Y56R sequences are from [5]. The smURFP Y56F and smURFP Y56R sequences are missing two amino acids in the C-terminal linker before the hexahistidine-tag and are highlighted in red.

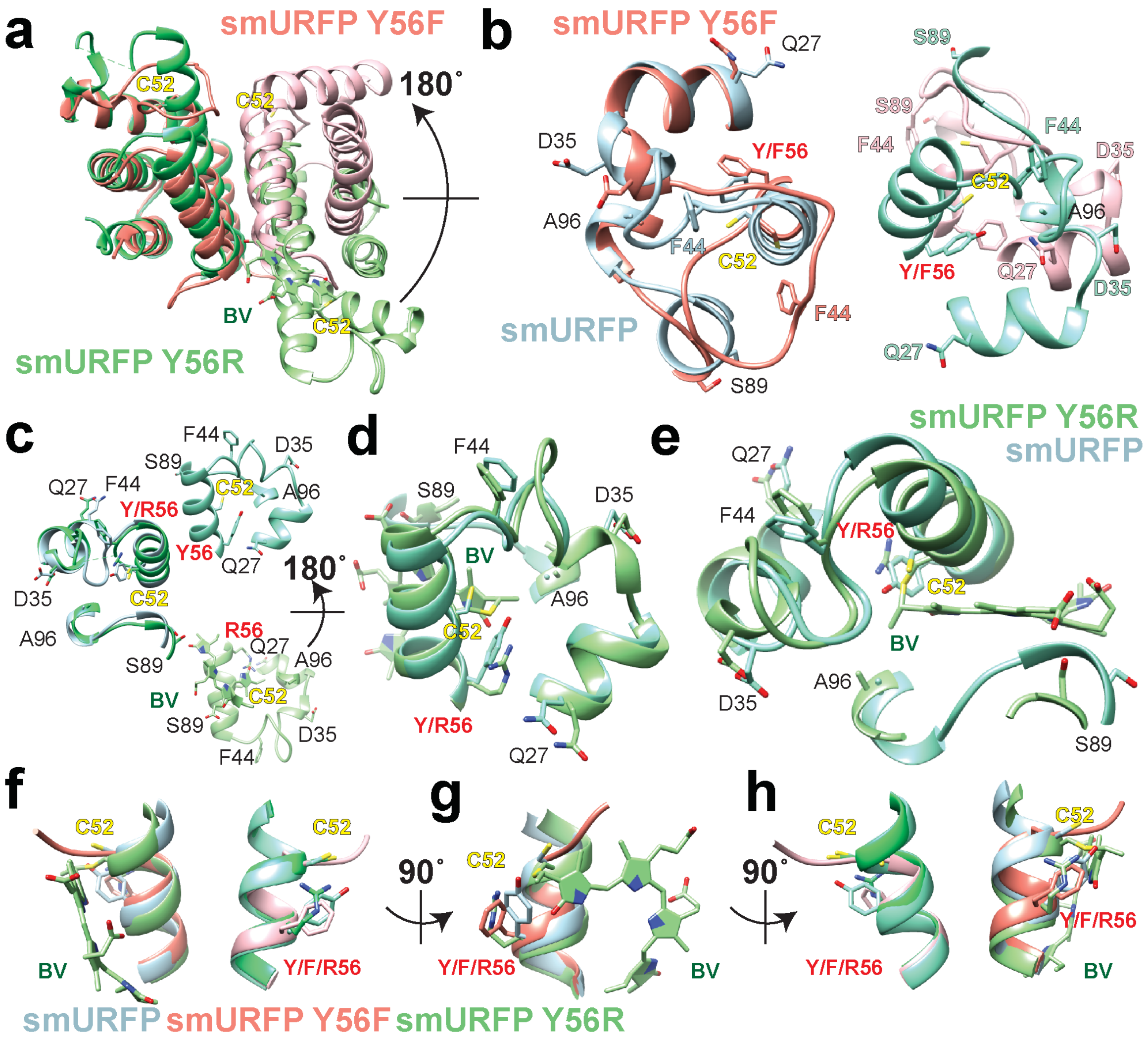

Supplementary Fig. 15 Comparison of smURFP to mutant crystal structures. The smURFP (blue), smURFP Y56R (green), and smURFP Y56F (pink) crystal structure alignments. **a** The smURFP Y56R (green) right protomer is rotated ~180˚ relative to the smURFP Y56F (pink) protomer. **b** Structural alignment of the smURFP (blue) and smURFP Y56F (pink) structures. Key amino acids in the chromophore pocket are labeled. C52 (yellow) covalently attaches BV. The smURFP Y56F pocket is flexible with disordered loops. smURFP shows a pre-formed chromophore pocket and a rigid structure. **c** The alignment of the smURFP (blue) and smURFP Y56R (green) crystal structures show a ~180˚ rotation in the right protomer. **d & e** The right protomer of smURFP Y56R was rotated 180˚ to compare structures. **d** smURFP and smURFP Y56R align well in the right protomer. **e** Top view of the chromophore pocket of smURFP and smURFP Y56R with BV. **f-h** Alignment of the three structures showing C52 (yellow) and position 56. The α-helices adopt a similar conformation. Position 56 is <4 Å from C52 attached to BV and directly influences the photophysical properties.

Supplementary Table 1 Data collection and refinement statistics of the smURFP crystal structure.

	smURFP (7UQA)
**Data collection**	
Space group	$C222_1$
Cell dimensions	
a, *b*, *c* (Å)	81.09, 141.80, 171.94
α, β, γ (°)	90.0, 90.0, 90.0
Resolution (Å)	99.00 – 2.80 (2.90 – 2.80) *
R_{merge}	0.14 (1.19)
R_{meas} (%)	16.20 (139.00)
R_{pim} (%)	8.00 (69.90)
$I / \sigma I$	9.99 (1.25)
Completeness (%)	99.5 (99.8)
Redundancy	3.9 (3.6)
**Refinement**	
Resolution (Å)	44.57 – 2.80 (2.91 – 2.80)
No. reflections	24,591
R_{work} / R_{free}	0.239/0.263
No. atoms	6,172
Protein	6,076
Ion (Cl^- / Na^+)	14 / 1
Water	81
B-factors	
Average B-factors (Å^2)	66.2
Protein	66.3
Ion	72.0
Water	57.5
R.m.s. deviations	
Bond lengths (Å)	0.003
Bond angles (°)	0.62

*Values in parentheses are for the highest-resolution shell.

Supplementary Table 2 Summary of the smURFP directed evolution with mutation locations & photophysical properties.

Fluorescent Protein	New Mutations	Mutation Locations	Directed Evolution Selection	PCB Incorp	BV Incorp
TeAPCα				No	No
R1+PCB	N42I	Chr. Pocket	**1) PCB Incorporation**	Yes	No
R2-1+PCB	Y65F, G66C	Dim. Interf.		Yes	No
R3-2+PCB	V83I, V98M	Chr. Pocket, Ext.		Yes	No
RS_R4-1	G45S, G57R, R61H, Q129K	Ext., Dim. Interf., Chr. Pocket, Ext.	**2) BV Incorporation**	Yes	Yes
RS_R5-2	G4C, Y56H	Ext., Chr. Pocket		Yes	Yes
R6-6	F36L, **H56Y**, **R57G**, D73G	Chr. Pocket, Ext.	**3) Blueshift Fluorescence**	Yes	Yes
R7-7	E18K, R33H, M131I	Salt Bridges, Ext.		Yes	Yes
R8-8	E48D, G96A, K118N, **I131M**	Ext., Chr. Pocket, Ext.	**4) Enhance Expression**	Yes	Yes
R8-9	K9N, **G96D**	Ext., Chr. Pocket		Yes	Yes
R10-10	S15T	Chr. Pocket		Yes	Yes
R11-2	G82S, D113E	Chr. Pocket, Ext.		Yes	Yes
smURFP	**C4S**, Y59F, **E113D**	Ext., Chr. Pocket, Ext.		Yes	Yes

Fluorescent Protein	Exc Max (nm)	Em Max (nm)	EC (M^{-1} cm^{-1})	EC % Change	QY (%)	QY % Change	Net Charge	Molecular Brightness Relative to smURFP (%)	Molecular Brightness % Change	FP Brightness in Bacteria Relative to R1+PCB
TeAPCα	NA	NA	NA	NA	NA	NA	-2.1	NA	NA	NA
R1+PCB	ND	ND	ND	NA	ND	NA	-2.1	ND	NA	1
R2-1+PCB	626	648	65,000	NA	7.2	NA	-2.1	14	NA	30
R3-2+PCB	620	648	74,000	14	13	81	-2.1	30	106	50
RS_R4-1	647	674	93,000	26	9.0	-31	-1	26	-13	50
RS_R5-2	671	696	71,000	-24	5.3	-41	-1	12	-55	50
R6-6	648	676	190,000	168	9.6	81	-2.1	56	385	50
R7-7	648	676	250,000	32	16	67	0	123	119	65
R8-8	648	672	260,000	4	13	-19	-1	104	-16	310
R8-9	644	672	175,000	-33	12	-8	-1.1	65	-38	295
R10-10	646	672	200,000	14	15	25	-2	93	43	380
R11-2	642	672	190,000	-5	12	-20	-2	70	-24	418
smURFP	642	670	180,000	-5	18	50	-1.9	100	42	650

The directed evolution of the FPs and photophysical values are from Reference [3].

New Mutations are different mutations relative to the previous round of directed evolution, or row.
Red is a mutation that reverted to the TeAPCα protein sequence. **Purple** is a mutation not kept in a subsequent round of directed evolution. **Green** is a mutation made to avoid disulfide bond formation.

The **Mutation Locations** on the smURFP structure are shown in Supplementary Figs. 4-6.

Directed Evolution Selection:
1) PCB Incorporation – Contained HO-1 & PcyA for PCB production in bacteria. Exc/Em = 685/710LP nm filters.
2) BV Incorporation – Contained HO-1 for BV production in bacteria. Exc/Em = 685/710LP nm filters.
3) Blueshift Fluorescence – Exc/Em = 650/690 nm filters.
4) Enhance Expression – Colonies picked at room temperature after 1-3 weeks. Exc/Em = 650/690 nm filters.

The **Net Charge** was calculated with Innovagen Protein Calculator (http://pepcalc.com/protein-calculator.php).

All percent changes are relative to the previous row. The yellow highlight is the largest value in a column.
Abbreviations: Chr. – chromophore; Dim. Interf. – dimeric interface; Ext. – exterior; Incorp. – incorporation; Exc – excitation; Em – emission; EC – extinction coefficient; % - percent; QY – quantum yield; LP – long pass

Supplementary Table 3 smURFP, smURFP Y56R, and smURFP Y56F chromophore pocket and total protein volumes.

Fluorescent Protein	Chromophore Pocket 1 Volume (Å^3)	Chromophore Pocket 2 Volume (Å^3)	Total Protein Volume (nm^3)
smURFP	576	213	31.4
smURFP Y56R	646	271	32.3
smURFP Y56F	663	442	33.2

We calculated pocket and total protein volumes as described in the Methods section.

Each FP homodimer contains two chromophore pockets. One cavity is larger to bind and subsequently attach BV, while the second cavity is smaller and must expand to allow the second BV for FP saturation. We previously showed that smURFP could covalently incorporate two BV by biophysical characterization and mass spectrometry[3]. In this work, we show smURFP with two BV attached by mass spectrometry (Supplementary Fig. 1d) and MD simulations (Fig. 2f, Supplementary Movies 3, 4, and Supplementary Structures 1, 2).

Supplementary References

1. Srinivasan, J., Cheatham, T. E., Cieplak, P., Kollman, P. A. & Case, D. A. Continuum solvent studies of the stability of DNA, RNA, and phosphoramidate - DNA helices. *J. Am. Chem. Soc.* **120**, 9401-9409 (1998).
2. Shang, L., Rockwell, N. C., Martin, S. S. & Lagarias, J. C. Biliverdin amides reveal roles for propionate side chains in bilin reductase recognition and in holophytochrome assembly and photoconversion. *Biochemistry* **49**, 6070-6082 (2010).
3. Rodriguez, E. A., Tran, G. N., Gross, L. A., Crisp, J. L., Shu, X., Lin, J. Y. & Tsien, R. Y. A far-red fluorescent protein evolved from a cyanobacterial phycobiliprotein. *Nat. Methods* **13**, 763-769 (2016).
4. Sonani, R. R., Gupta, G. D., Madamwar, D. & Kumar, V. Crystal structure of allophycocyanin from marine cyanobacterium *Phormidium* sp. A09DM. *PLoS One* **10**, e0124580 (2015).
5. Fuenzalida-Werner, J. P., Janowski, R., Mishra, K., Weidenfeld, I., Niessing, D., Ntziachristos, V. & Stiel, A. C. Crystal structure of a biliverdin-bound phycobiliprotein: Interdependence of oligomerization and chromophorylation. *J. Struct. Biol.* **204**, 519-522 (2018).
6. Machado, J.-H., Ting, R., Lin, J. Y. & Rodriguez, E. A. A self-labeling protein based on the small ultra-red fluorescent protein, smURFP. *RSC Chem. Biol.* **2**, 1221-1226 (2021).
