# Supplementary material for: Structural and photophysical characterization of the small ultra-red fluorescent protein
Source: Nat Commun. 2023 Jul 12;14:4155. doi: 10.1038/s41467-023-39776-9 (PMC10338489; doi:10.1038/s41467-023-39776-9)
Supplement: Supplementary file 2 — Description of additional supplementary files [file 41467_2023_39776_MOESM2_ESM.pdf]

Description of additional supplementary files

Supplementary Movie 1 The smURFP crystal structure. The asymmetric unit contains three smURFP homodimers in the $C222_1$ space group with 6,172 atoms. At the center is a sodium ion coordinated by Q46 on each smURFP protomer. The sodium (Na+) ion is violet, chloride (Cl-) ions are green, and water is red. Chains A, C, and E are cyan, and chains B, D, and F are light green.

Supplementary Movie 2 The smURFP hydrogen bonds. The structure contains 295 hydrogen bonds, including water. Three hydrogen bonds and four salt bridges stabilize the homodimeric interface. Hydrogen bonds are dotted lines, while nitrogen, oxygen, and sulfur are colored blue, red, and yellow, respectively. Chain E is cyan, and chain F is light green.

Supplementary Movie 3 Superimposition of smURFP+BV and smURFP+BV_2. Docking and MD simulations placed one and two BV molecules into our smURFP structure, as described in the Methods section. smURFP+BV and smURFP+BV_2 are cyan and green, respectively. The smURFP+BV_2 structure must expand the unoccupied pocket to allow entry and incorporation of the second BV. The smURFP accommodated two BV molecules without significant rearrangement of the homodimeric structure.

Supplementary Movie 4 The smURFP+BV_2 with protein surface. Docking and MD simulations placed two BV molecules into our smURFP structure, as described in the Methods section. The smURFP protein surface is gray. The two BV molecules are dark green, with oxygen and hydrogen colored red and white. The smURFP accommodated two BV molecules without significant rearrangement of the homodimeric structure. The carboxylates are exposed to solvent, as expected from incorporating chemically modified BV analogues[6].

Supplementary Movie 5 The smURFP homodimer interface. The dimeric interface of the smURFP with H61, F65, and I78 residues in red, with nitrogen in blue. C52 is shown with sulfur in yellow. Chain E is cyan, and chain F is light green.

Supplementary Movie 6 Comparison of smURFP to mutant crystal structures. smURFP (blue), smURFP Y56R (green), and smURFP Y56F (pink) are aligned from residues 50-58. Position 56 is directly positioned near C52 and BV to define the pocket and rigidize BV. smURFP Y56F is unstructured at the top of the α-helix. BV from smURFP Y56R (PDB ID: 6FZN [https://doi.org/10.2210/pdb6FZN/pdb]) is dark green, while nitrogen, oxygen, and sulfur are colored blue, red, and yellow, respectively.
