# Supplementary material for: Structural and photophysical characterization of the small ultra-red fluorescent protein
Source: Nat Commun. 2023 Jul 12;14:4155. doi: 10.1038/s41467-023-39776-9 (PMC10338489; doi:10.1038/s41467-023-39776-9)
Supplement: Supplementary file 9 — Reporting Summary [file 41467_2023_39776_MOESM9_ESM.pdf]

nature portfolio

Corresponding author(s): Erik A. Rodriguez

Last updated by author(s): Jun 13, 2023

Reporting Summary

Nature Portfolio wishes to improve the reproducibility of the work that we publish. This form provides structure for consistency and transparency in reporting. For further information on Nature Portfolio policies, see our Editorial Policies and the Editorial Policy Checklist.

Statistics

For all statistical analyses, confirm that the following items are present in the figure legend, table legend, main text, or Methods section.

n/a	Confirmed	
☐	☒	The exact sample size (*n*) for each experimental group/condition, given as a discrete number and unit of measurement
☐	☒	A statement on whether measurements were taken from distinct samples or whether the same sample was measured repeatedly
☐	☒	The statistical test(s) used AND whether they are one- or two-sided<br>*Only common tests should be described solely by name; describe more complex techniques in the Methods section.*
☒	☐	A description of all covariates tested
☒	☐	A description of any assumptions or corrections, such as tests of normality and adjustment for multiple comparisons
☐	☒	A full description of the statistical parameters including central tendency (e.g. means) or other basic estimates (e.g. regression coefficient) AND variation (e.g. standard deviation) or associated estimates of uncertainty (e.g. confidence intervals)
☐	☒	For null hypothesis testing, the test statistic (e.g. *F*, *t*, *r*) with confidence intervals, effect sizes, degrees of freedom and *P* value noted<br>*Give P values as exact values whenever suitable.*
☒	☐	For Bayesian analysis, information on the choice of priors and Markov chain Monte Carlo settings
☒	☐	For hierarchical and complex designs, identification of the appropriate level for tests and full reporting of outcomes
☒	☐	Estimates of effect sizes (e.g. Cohen's *d*, Pearson's *r*), indicating how they were calculated

Our web collection on statistics for biologists contains articles on many of the points above.

Software and code

Policy information about availability of computer code

Data collection	The crystal diffraction data were indexed, integrated, and scaled using HKL-2000 V. 715.5. Molecular replacement was performed using Phaser V. 2.8.2 in the CCP4 program suite V. 7.0.051. Model building and refinement were manually performed using the programs Coot V. 0.8.9.2 and Phenix V. 1.12_2829, respectively. Raw images were collected with Zeiss Zen Blue V. 2.3, ThermoFisher EVOS FL Auto 2 V.2.0.2094.0, and Leica Application Suite X V. 3.5.7.23225 software. Two-photon absorption spectrum and absolute cross-sections of smURFP used a LabVIEW V. 2018 program to automatically tune an Insight DeepSee (Spectra-Physics) femtosecond laser for fluorescence excitation. References are in the manuscript.
Data analysis	The mass spectra were deconvolved using ProMassCalc or Extract_MSN in Xcalibur V. 2.2 software (ThermoFisher). MolProbity V. 4.02b-467 validated the smURFP structure. The Schrödinger V. 2021.2 and Desmond V. 6.6 software packages performed MD simulations using our smURFP structure. 3V: Voss Volume Voxelator V. 1.3 created internal cavities. UCSF Chimera Software V. 1.16 or X 1.5 measured chromophore cavities and protein volume, created structure figures, and made movies. PyMOL V. 1.8.6.2 calculated the electrostatic potential and hydrophobicity surfaces using the APBS plugin and Color_h script. EMBL-EBI Clustal Omega V. 1.2.4 (https://www.ebi.ac.uk/Tools/msa/clustalo/) aligned protein sequences and MView V. 1.63 determined the consensus sequence and percent identity. ImageJ (Fiji) V. 2.3.0/1.53t analyzed epifluorescence images. Celleste Image Analysis Software V 5.0.2.6993 (ThermoFisher) deconvolved OSER Assay images. KaleidaGraph V. 5.0.4 (Synergy Software) calculated statistics. The protein Net Charge was calculated with Innovagen Protein Calculator (http://pepcalc.com/protein-calculator.php, no version number listed) in Supplementary Table 2. References are in the manuscript.

For manuscripts utilizing custom algorithms or software that are central to the research but not yet described in published literature, software must be made available to editors and reviewers. We strongly encourage code deposition in a community repository (e.g. GitHub). See the Nature Portfolio guidelines for submitting code & software for further information.

Data

Policy information about availability of data

All manuscripts must include a data availability statement. This statement should provide the following information, where applicable:

- Accession codes, unique identifiers, or web links for publicly available datasets
- A description of any restrictions on data availability
- For clinical datasets or third party data, please ensure that the statement adheres to our policy

Data Availability
The smURFP crystal structure is available at the Protein Data Bank under accession code 7UQA [https://doi.org/10.2210/pdb7UQA/pdb]. The following crystal structures were used in this study, Protein Data Bank accession codes 4RMP [https://doi.org/10.2210/pdb4RMP/pdb]39, 6FZN [https://doi.org/10.2210/pdb6FZN/pdb]28, 6FZO [https://doi.org/10.2210/pdb6FZO/pdb]28, and 4PO5 [https://doi.org/10.2210/pdb4PO5/pdb]47. GenBank/EMBL/DDBJ ascension codes are KX449134 [https://www.ncbi.nlm.nih.gov/nuccore/KX449134] and KX449135 [https://www.ncbi.nlm.nih.gov/nuccore/KX449135] for smURFP and TDsmURFP, respectively. Plasmid DNA for smURFP and TDsmURFP for bacterial and mammalian expression is available from Addgene (80341, 80342, 80343, 80344) [https://www.addgene.org/Erik_Rodriguez/]. The source data for Figs. 3d, 4a-b, 5, and Supplementary Figs. 3a-c, 7e-f are provided in the Excel Source Data file with this paper. Additionally, the smURFP structures with one and two BV (Supplementary Structures 1, 2) are provided in the Source Data file.

Human research participants

Policy information about studies involving human research participants and Sex and Gender in Research.

Reporting on sex and gender	Not applicable.
Population characteristics	Not applicable.
Recruitment	Not applicable.
Ethics oversight	Not applicable.

Note that full information on the approval of the study protocol must also be provided in the manuscript.

Field-specific reporting

Please select the one below that is the best fit for your research. If you are not sure, read the appropriate sections before making your selection.

☒ Life sciences ☐ Behavioural & social sciences ☐ Ecological, evolutionary & environmental sciences

For a reference copy of the document with all sections, see nature.com/documents/nr-reporting-summary-flat.pdf

Life sciences study design

All studies must disclose on these points even when the disclosure is negative.

Sample size	No sample-size calculations were performed. Sample sizes were similar or greater to those generally employed in the field. For gels of fluorescent proteins refer to Rodriguez et al. Nature Methods 13(9) 763-9 (2016). For comparison of fluorescent proteins in mammalian cells refer to Yu, D. et al. Nature Communications 5, 3626 (2014), and Rodriguez et al. Nature Methods 13(9) 763-9 (2016). For the OSER Assay refer to Costantini, L. M. et al. Traffic 13, 643-649 (2012). For single-molecule imaging refer to Saurabh, S. et al. J. Am. Chem. Soc. 138, 10398-10401 (2016).
Data exclusions	OSER Assay cells exhibiting excessive brightness and an abnormal phenotype (multiple nuclei, lobed nucleus, disorganized ER membrane, or undefined nuclear envelope) were not analyzed. Saturated cell fluorescence was not analyzed. All exclusion criteria were pre-determined.
Replication	The replication, including sample size, number of cells, number of gels, number of single-molecules, or other unit, are indicated in the Figure legends. All replication attempts were successful.
Randomization	HEK293A cells were randomly seeded into dishes before transfection. Cell location was randomly chosen for acquiring images.
Blinding	We did not perform blinding because experimental conditions were known to the researcher to add appropriate molecules and fluorescently image with proper filters. Transfected cells were analyzed using an unbiased statistical test. Unbiased, quantitative data was obtained without subjective classifications of random cells.

Reporting for specific materials, systems and methods

We require information from authors about some types of materials, experimental systems and methods used in many studies. Here, indicate whether each material, system or method listed is relevant to your study. If you are not sure if a list item applies to your research, read the appropriate section before selecting a response.

Materials & experimental systems

n/a	Involved in the study
[x]	[ ] Antibodies
[ ]	[x] Eukaryotic cell lines
[x]	[ ] Palaeontology and archaeology
[x]	[ ] Animals and other organisms
[x]	[ ] Clinical data
[x]	[ ] Dual use research of concern

Methods

n/a	Involved in the study
[x]	[ ] ChIP-seq
[x]	[ ] Flow cytometry
[x]	[ ] MRI-based neuroimaging

Eukaryotic cell lines

Policy information about cell lines and Sex and Gender in Research

Cell line source(s)	ThermoFisher supplied the HEK293A cell line (ThermoFisher R70507).
Authentication	ThermoFisher verified the HEK293A cell line (ThermoFisher R70507) identity. The HEK293A cells were maintained in isolation to avoid cross-contamination. The flat HEK293A cell morphology confirmed the cellular identity of HEK293A cells before transfection and cellular imaging.
Mycoplasma contamination	Cells were tested regularly to be free of mycoplasma contamination with DNA staining.
Commonly misidentified lines (See ICLAC register)	HEK cells are listed by the ICLAC as a misidentified cell line often contaminated with HeLa cells, which are not used in our laboratory. HEK293A cell line was used in the experiments. However, the cell identity was not central to the outcome of these experiments.
